# Trigger vs. Substrate: Multi-Dimensional Modulation of QT-Prolongation Associated Arrhythmic Dynamics by a hERG Channel Activator

**DOI:** 10.3389/fphys.2017.00757

**Published:** 2017-10-04

**Authors:** Michael A. Colman, Erick A. Perez Alday, Arun V. Holden, Alan P. Benson

**Affiliations:** ^1^School of Biomedical Sciences and Multidisciplinary Cardiovascular Research Centre, University of Leeds, Leeds, United Kingdom; ^2^Division of Cardiovascular Medicine, Oregon Health and Science University, Portland, OR, United States

**Keywords:** QT interval, action potential duration, arrhythmia trigger, arrhythmia substrate, hERG activators, computational modeling

## Abstract

**Background:** Prolongation of the QT interval of the electrocardiogram (ECG), underlain by prolongation of the action potential duration (APD) at the cellular level, is linked to increased vulnerability to cardiac arrhythmia. Pharmacological management of arrhythmia associated with QT prolongation is typically achieved through attempting to restore APD to control ranges, reversing the enhanced vulnerability to Ca^2+^-dependent afterdepolarisations (arrhythmia triggers) and increased transmural dispersion of repolarisation (arrhythmia substrate) associated with APD prolongation. However, such pharmacological modulation has been demonstrated to have limited effectiveness. Understanding the integrative functional impact of pharmacological modulation requires simultaneous investigation of both the trigger and substrate.

**Methods:** We implemented a multi-scale (cell and tissue) *in silico* approach using a model of the human ventricular action potential, integrated with a model of stochastic 3D spatiotemporal Ca^2+^ dynamics, and parameter modification to mimic prolonged QT conditions. We used these models to examine the efficacy of the hERG activator MC-II-157c in restoring APD to control ranges, examined its effects on arrhythmia triggers and substrates, and the interaction of these arrhythmia triggers and substrates.

**Results:** QT prolongation conditions promoted the development of spontaneous release events underlying afterdepolarisations during rapid pacing. MC-II-157c applied to prolonged QT conditions shortened the APD, inhibited the development of afterdepolarisations and reduced the probability of afterdepolarisations manifesting as triggered activity in single cells. In tissue, QT prolongation resulted in an increased transmural dispersion of repolarisation, which manifested as an increased vulnerable window for uni-directional conduction block. In some cases, MC-II-157c further increased the vulnerable window through its effects on *I*_Na_. The combination of stochastic release event modulation and transmural dispersion of repolarisation modulation by MC-II-157c resulted in an integrative behavior wherein the arrhythmia trigger is reduced but the arrhythmia substrate is increased, leading to variable and non-linear overall vulnerability to arrhythmia.

**Conclusion:** The relative balance of reduced trigger and increased substrate underlies a multi-dimensional role of MC-II-157c in modulation of cardiac arrhythmia vulnerability associated with prolonged QT interval.

## Introduction

Conditions in which the QT interval of the electrocardiogram (ECG) is prolonged, such as heart failure (Hart, 1994) and inherited or acquired long QT syndromes (LQTS) (Schwartz et al., 2012), are associated with an increased risk of ventricular arrhythmias (Tomaselli and Zipes, 2004; Moss and Kass, 2005). The prolonged QT interval reflects prolongation of the ventricular cellular action potential duration (APD), which can result in arrhythmias through an increase in cell-level arrhythmia triggers and/or modification of tissue-level arrhythmia substrates (Kalin et al., 2010; Benson et al., 2011a; Tse, 2016).

Cell-level triggers arise because delayed ventricular repolarisation modifies membrane and subcellular Ca^2+^ handling (Clusin, 2003; Němec et al., 2016), leading to either re-activation of the L-type Ca^2+^ current and early afterdepolarisations (Lankipalli et al., 2005), or Ca^2+^ overload of the sarcoplasmic reticulum (SR), causing spontaneous SR Ca^2+^ release events and delayed-afterdepolarisations through activation of the forward-mode Na^+^-Ca^2+^ exchange current (*I*_NaCa_), which in turn can result in triggered activity if the delayed afterdepolarisation is of sufficient magnitude (Janse, 2004). Cardiomyocytes typically exhibit a threshold dependence of the occurrence of spontaneous release events as a function of SR Ca^2+^ load, wherein the probability of a spontaneous release event rapidly rises from 0 to 1 within a critical region of SR Ca^2+^ (Venetucci et al., 2007; Campos et al., 2015). Although originating at the cell level, triggers need to be coordinated at the tissue level in order to develop into arrhythmias: a critical compact region of tissue simultaneously exhibiting triggered behavior is required to initiate propagation of the trigger (Noble, 1972; Clayton et al., 2011; Bezekci et al., 2015; Campos et al., 2015).

An increase in the tissue-level substrates for arrhythmias (that is, the necessary conditions for triggered activity to propagate and develop into arrhythmias) arise because APD prolongation is rarely homogenous in and between the different regions of the ventricles (e.g., transmurally, or from base to apex) (Antzelevitch, 2005; Glukhov et al., 2010). This heterogeneous APD prolongation increases the spatial dispersion of repolarisation, potentially leading to regions of recovered (i.e., excitable) tissue partially bordered by still refractory (i.e., unexcitable) tissue. A propagating trigger event occurring in such a location can be partially blocked by the refractory tissue, leading to re-entrant arrhythmias (Pandit and Jalife, 2013). The spatiotemporal region where such partial conduction block could occur is termed the “vulnerable window” (VW) (Starmer et al., 1993; Shaw and Rudy, 1995; Benson et al., 2008, 2011a). It follows that heterogeneous APD prolongation increases not only the spatial dispersion of repolarisation, but also the VW, i.e., the arrhythmia substrate.

The interaction of triggers and substrates determines the initiation of arrhythmia: an arrhythmia cannot be initiated without both a suitably-sized and -timed trigger and the necessary substrate to allow that trigger to propagate in a re-entrant manner (Kalin et al., 2010). Arrhythmias, therefore, are not cellular events, but tissue-level events.

Management of arrhythmias associated with QT prolongation can be achieved by attempting to restore the ventricular APD to control ranges (Nachimuthu et al., 2012), thus reversing the increases in arrhythmogenic triggers and substrates associated with APD prolongation. One such strategy is the use of human ether-a-go-go-related-gene channel (hERG) activators that enhance the repolarising rapid delayed rectifier K^+^ current (*I*_Kr_), thus reducing the APD (Grunnet et al., 2008; Wu and Sanguinetti, 2016). However, many anti-arrhythmic drugs have pro-arrhythmic effects (Kumar and Zimetbaum, 2013); such drugs can shorten the action potential and the QT interval (reducing arrhythmias associated with a prolonged QT interval), but they may have additional and unintended effects that increase (rather than reduce) the propensity for arrhythmias under certain conditions. For example, we have shown in a previous experimental and computational study that the hERG activator NS1643, one of the most effective and best characterized hERG activators (Hansen et al., 2006), successfully restores APD toward healthy durations and reduces arrhythmia triggers, but is associated with an increase in the VW, i.e., the substrate for arrhythmias, due to effects on the post-repolarisation refractory period (Peitersen et al., 2008). Furthermore, while low concentrations of NS1643 activates *I*_Kr_ and shortens APD, it has been shown that higher concentrations of NS1643 blocks (rather than activates) *I*_Kr_ (Bilet and Bauer, 2012).

The potentially pro-arrhythmic effects of hERG activators, such as NS1643 has prompted the search for novel hERG activators that do not display these effects. One recently-identified compound is MC-II-157c, an NS1643 analog. MC-II-157c activates *I*_Kr_ at low concentrations and, unlike NS1643, it continues to activate *I*_Kr_ at high concentrations; it may also block the sodium current (*I*_Na_) (Guo et al., 2014). However, it remains unknown how the *I*_Kr_ activation and *I*_Na_ block seen with this new compound affect arrhythmia triggers and substrates, and importantly, how these (increased or decreased) triggers and substrates interact to induce arrhythmias (if at all).

Computational models provide powerful research tools to understand the intricacies of arrhythmia trigger and substrate interaction, as they allow us to modify parameters under precisely controlled conditions and quantify the resultant tissue-level arrhythmic behavior, and can predict how these arrhythmias will manifest in a clinical setting (e.g., changes to the ECG). We therefore used a computational approach to quantify the interaction of pharmacologically-modified arrhythmia trigger and substrate, using the novel hERG activator MC-II-157c as an example.

We wanted to quantify the modified triggers and substrate that result from MC-II-157c ion channel actions. To this end, we use detailed single cell and tissue level models to study the effect of QT prolongation and its modulation by MC-II-157c on: (i) APD heterogeneity in isolated cells; (ii) SR Ca^2+^ loading and subsequent spontaneous SR Ca^2+^ release events; (iii) the probability of spontaneous SR Ca^2+^ release manifesting as triggered action potentials in single cell and ectopic activity in tissue; and (iv) the vulnerability to the initiation of re-entrant like conduction patterns.

## Methods

We implemented a multi-scale *in silico* approach to study the interactions between arrhythmia trigger and substrate in conditions associated with prolonged QT intervals, and their modulation by the hERG activator MC-II-157c.

### Isolated cell models – intracellular Ca^2+^ handling

**Figure 1:**
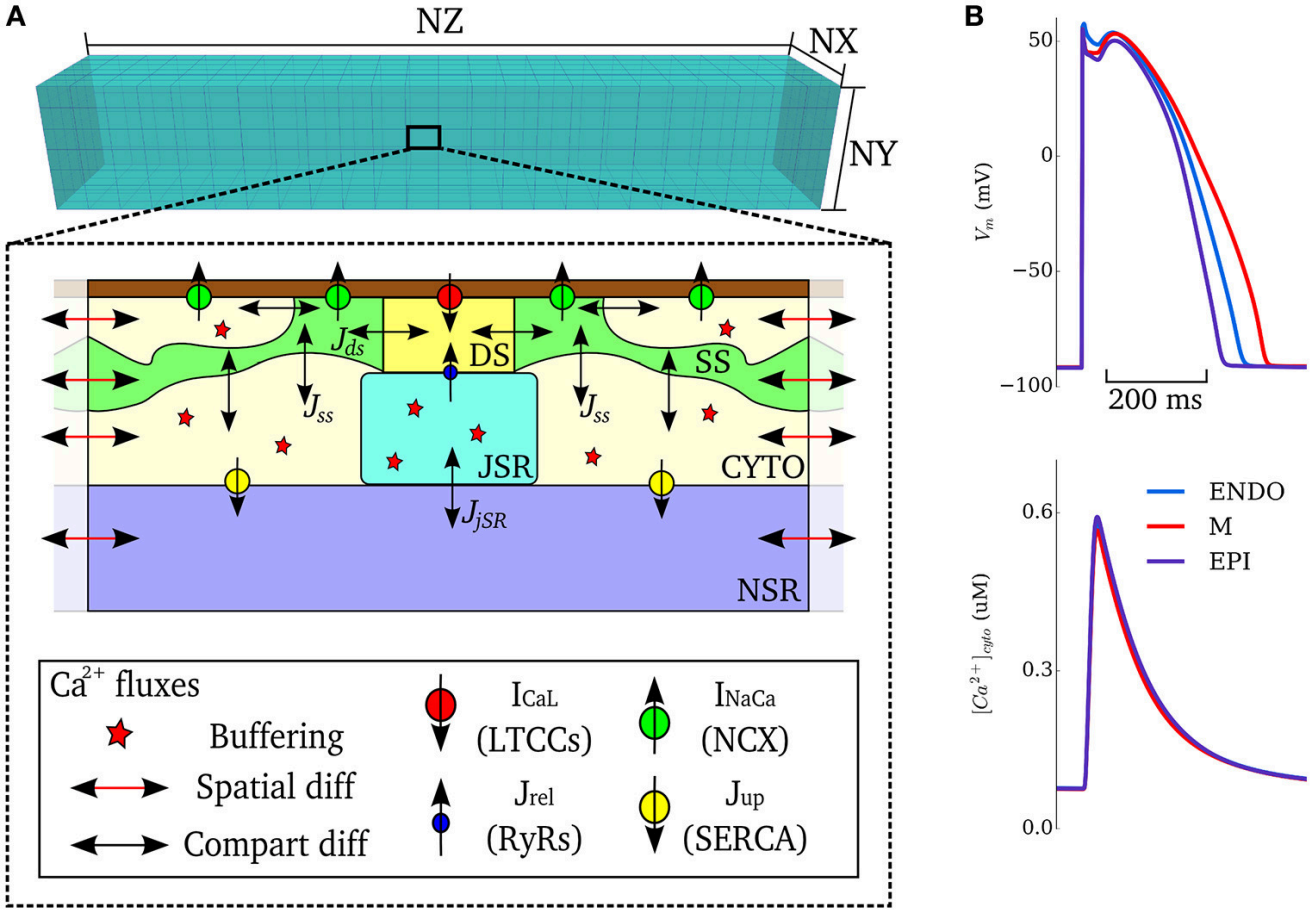
Model schematic. **(A)** Schematic structure of the spatio-temporal Ca^2+^ handling model with the compartments comprising a single CRU, and coupling between compartments, illustrated. DS, dyadic cleft space; SS, subspace; CTYO, bulk intracellular space; JSR, junctional SR; NSR, network SR. NX, 15; NY, 20; NZ, 65. **(B)** Action potentials (upper) and Ca^2+^ transients (lower) during control pacing (BCL = 1, 000 ms) for the three cell types (ENDO, Endocardium; M, Midmyocardium; EPI, Epicardium).

In order to simulate triggered and ectopic activity underlain by spontaneous Ca^2+^ release events, a spatial model of intracellular Ca^2+^ handling which explicitly accounts for stochastic state transitions and spatial coupling is required. We therefore implemented an efficient, idealized reduction of a previously developed and validated general model of spatio-temporal Ca^2+^ handling with realistic structure (Colman et al., 2017), using a similar approach implemented by other groups (e.g., Restrepo et al., 2008). Briefly, 15 × 20 × 65 spatially-discrete individual calcium release units (CRUs) were modeled throughout the geometry of the cell (Figure 1). Each CRU comprises of five compartments with associated Ca^2+^ concentrations: the intracellular spaces of the dyadic cleft space ([Ca^2+^]_ds_), subspace ([Ca^2+^]_SS_) and bulk-cytosolic space ([Ca^2+^]_i_), and the network and junctional SR ([Ca^2+^]_nSR_, ([Ca^2+^]_jSR_). The bulk cytosol, subspace and network SR are diffusively coupled to neighboring CRUs; the dyadic cleft space and junctional SR are not spatially coupled to neighbors. The fundamental model equations describing this system are:

$$\begin{array}{l} \frac{\text{d}{\left[\text{C}{\text{a}}^{2+}\right]}_{\text{i}}}{\text{dt}}=\beta \left(\text{D}\nabla^{2}{\left[\text{C}{\text{a}}^{2+}\right]}_{\text{i}}+\varphi_{\text{i}}+\left(\nu_{\text{SS}}/\nu_{\text{i}}\right)J_{\text{SS}}\right)\\ \frac{\text{d}{\left[\text{C}{\text{a}}^{2+}\right]}_{\text{SS}}}{\text{dt}}=\text{D}\nabla^{2}{\left[\text{C}{\text{a}}^{2+}\right]}_{\text{SS}}+\varphi_{\text{SS}}-J_{\text{SS}}+\left(\nu_{\text{ds}}/\nu_{\text{SS}}\right)J_{\text{ds}}\\ \frac{\text{d}{\left[\text{C}{\text{a}}^{2+}\right]}_{\text{nSR}}}{\text{dt}}=\beta_{\text{nSR}}\left(\text{D}\nabla^{2}{\left[\text{C}{\text{a}}^{2+}\right]}_{\text{nSR}}+\varphi_{\text{nSR}}-\left(\nu_{\text{jSR}}/\nu_{\text{nSR}}\right)J_{\text{jSR}}\right)\\ \frac{\text{d}{\left[\text{C}{\text{a}}^{2+}\right]}_{\text{ds}}}{\text{dt}}=\varphi_{\text{ds}}-J_{\text{ds}}\\ \frac{\text{d}{\left[\text{C}{\text{a}}^{2+}\right]}_{\text{jSR}}}{\text{dt}}=\varphi_{\text{jSR}}-{\text{J}}_{\text{jSR}} \end{array}$$

where transfer between compartments is given by:

$$\begin{array}{l} J_{\text{SS}}=\left({\left[\text{C}{\text{a}}^{2+}\right]}_{\text{SS}}-{\left[\text{C}{\text{a}}^{2+}\right]}_{\text{i}}\right)\tau_{\text{SS}}^{-1}\\ J_{\text{ds}}=\left({\left[\text{C}{\text{a}}^{2+}\right]}_{\text{ds}}-{\left[\text{C}{\text{a}}^{2+}\right]}_{\text{SS}}\right)\tau_{\text{ds}}^{-1}\\ J_{\text{jSR}}=\left({\left[\text{C}{\text{a}}^{2+}\right]}_{\text{jSR}}-{\left[\text{C}{\text{a}}^{2+}\right]}_{\text{nSR}}\right)\tau_{\text{jSR}}^{-1} \end{array}$$

where *v*_*x*_ is the volume of compartment *x*, β_*x*_ is an instantaneous buffering term, and φ_*x*_ is the reaction term. Stochastic dynamics are modeled for the RyRs and LTCCs, which are part of the dyadic cleft space reaction term. All parameters and reaction terms are given in the Supplementary Material.

### Isolated cell models - action potential model

Ion currents were described by a simplified version of the O'Hara-Rudy dynamic (ORd) human ventricular cell model (O'Hara et al., 2011), wherein the major currents only (*I*_Na_, *I*_Kr_, *I*_Ks_, *I*_K1_, *I*_to_, *I*_NaK_) were included, without the further details of phosphorylation included in the original study; Ca^2+^ currents (*I*_CaL_, *I*_NaCa_, *I*_Cap_, *I*_Cab_) are described by the Ca^2+^ handling model. These simplifications were implemented to improve computational efficiency and for integration with the general stochastic Ca^2+^ handling model described above. The model provides specific formulations to describe the heterogeneity in ionic currents of the transmural cell types (endocardial, midmyocardial, and epicardial) found in the left ventricular free wall of the human heart (see O'Hara et al., 2011) for details.

This integrated stochastic framework captures spontaneous Ca^2+^ release events that could lead to triggered activity (see details in Simulating Spontaneous Release Events in Tissue Models below). Default model action potentials and cytosolic Ca^2+^ transients, for the three transmural cell types of the simplified ORd model with stochastic Ca^2+^ handling, are shown in Figure 1B. The updated cell model exhibits action potential and Ca^2+^ transient properties similar to the original cell model and within the range of experimental data presented in the original study (O'Hara et al., 2011): APD = 272–360 ms and intracellular Ca^2+^ transient magnitude of ~0.6 μM for the three cell-types during control pacing; note that the present study does not consider heterogeneity in the intracellular Ca^2+^ handling system and thus the Ca^2+^ transient is more homogeneous between the cell types than in the original study (see Limitations). The model is therefore considered suitable for the mechanistic study undertaken.

### Prolonged QT and pharmacological modulation

We were interested in general cases of QT prolongation rather than modeling the kinetics of very specific conditions, while still making our results broadly applicable to clinical conditions, such as LQTS. We therefore simulated QT interval prolongation (i.e., prolongation of the ventricular APD) in one of three ways: (i) downregulation of the slow delayed rectifier K^+^ current (*I*_Ks_) maximal conductance by 50%, similar to LQTS1, which we term “prolonged QT variant a” (PQTa) in the remainder of the manuscript; (ii) downregulation of *I*_Kr_ maximal conductance by 50% (PQTb), similar to LQTS2; and (iii) upregulation of *I*_Ca,L_ maximal conductance by 50% (PQTc), similar to LQT8 (Bohnen et al., 2017).

Effects of 10 μM of the hERG activator MC-II-157c on *I*_Kr_ were modeled by modifying the *I*_Kr_ formulation according to experimental data (Guo et al., 2014): maximal conductance was decreased by 12%, activation was shifted by −14 mV and inactivation by +14 mV, and deactivation kinetics were slowed 3.3-fold (note that, although the maximal conductance of *I*_Kr_ is reduced by MC-II-157c, its kinetic effects enhance the activity of the current; see The hERG Activator MC-II-157c Partially or Fully Reverses APD Prolongation Heterogeneously). Effects of MC-II-157c on blocking *I*_Na_ (which are not as well characterized as its effects on *I*_Kr_; Guo et al., 2014) were simulated by reducing the maximal conductance of *I*_Na_ by 0, 40, and 80%.

### Tissue models

We used a 20 mm 1D virtual tissue strand (Kléber and Rudy, 2004) for quantifying transmural propagation and vulnerability, with equal spatial distributions of endocardial, midmyocardial and epicardial cells. A 20 × 40 mm 2D tissue sheet (Clayton et al., 2011) was used for simulations examining propagation of triggered activity, with equal distributions of endocardial, midmyocardial and epicardial tissue in the *x* direction. For examining intramural propagation and ectopic activity in 3D, we used an anatomically detailed 3D ventricular wedge model, obtained by diffusion tensor MRI, and used equal proportions of endocardial, midmyocardial and epicardial tissue in the transmural direction (see Benson et al., 2007, 2011b; Walton et al., 2013 for details).

All tissues were isotropic, i.e., conduction velocity was set to be equal in all directions: We used an electrical diffusion coefficient of *D* = 0.048 mm^2^ms^−1^, to give a conduction time along the 1D strand (i.e., a transmural activation time) of 40 ms (cf. Glukhov et al., 2010), and a plane wave conduction velocity of 0.5 m.s^−1^ in all tissues. The body surface potential was computed by placing the ventricular wedge model in a human torso mesh; the forward problem was solved by a boundary element method, as has been described in previous studies (Perez Alday et al., 2015, 2016, 2017). ECGs were derived from the body surface potential by selecting elements of the torso mesh which correspond to the ECG electrodes.

### Simulating spontaneous release events in tissue models

Performing tissue level simulations using the fully detailed spatial Ca^2+^ handling model to describe individual cells is computationally intractable due to the large number of equations that need to be solved in such situations. Furthermore, the focus of this study was not to dissect the mechanisms of spontaneous Ca^2+^ release in single cell, but rather to understand the considerations determining the manifestation of sub-cellular Ca^2+^ release as triggered action potentials and propagating electrical excitation in the presence of prolonged APD and its pharmacological modulation.

**Figure 2:**
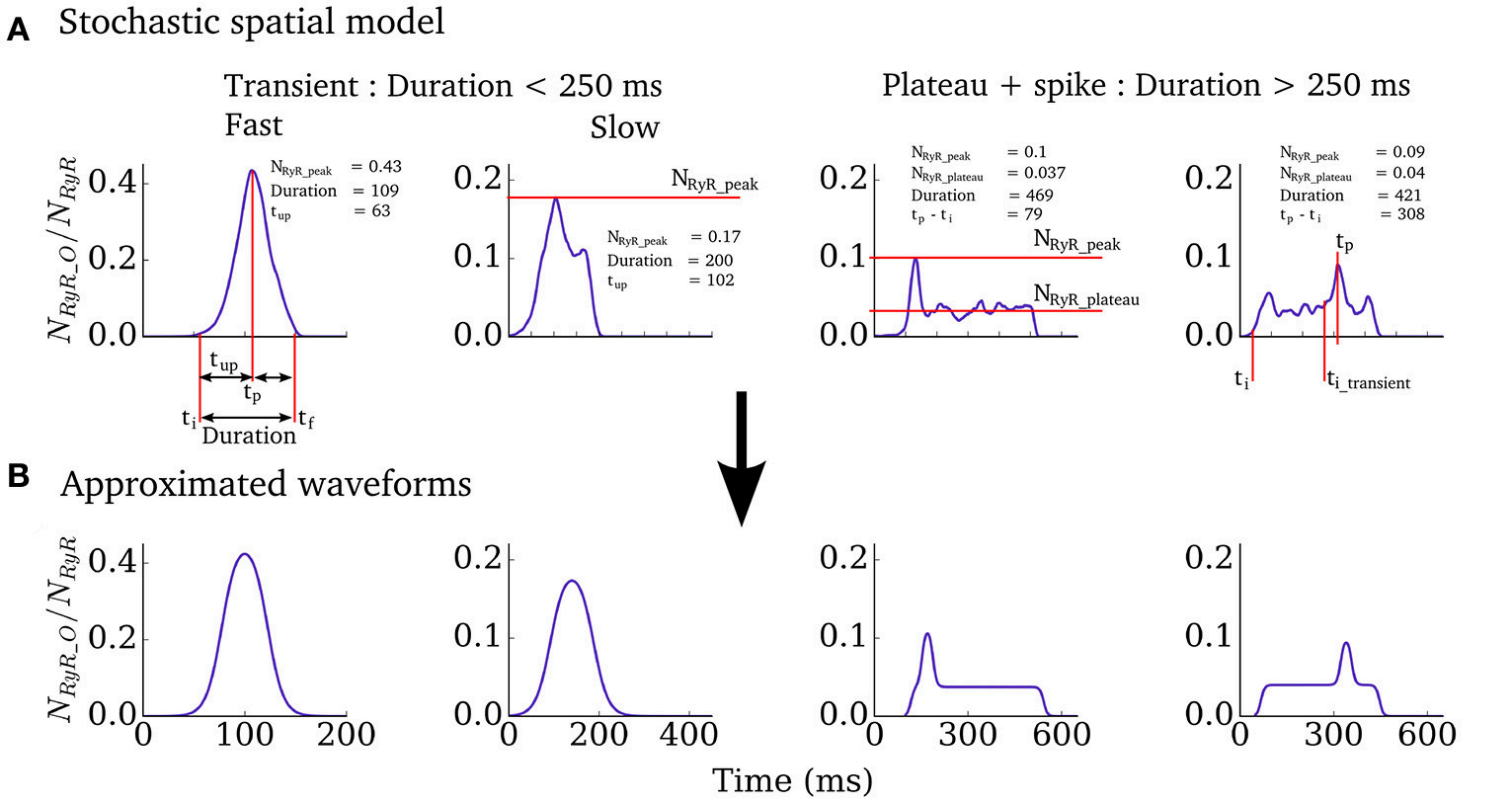
Spontaneous excitation RyR waveforms and parameters. **(A)** Four examples of RyR waveforms associated with whole-cell spontaneous release events in the spatial cell model. Parameters describing the shape of the waveform are labeled. **(B)** Analytical waveforms approximating those in **(A)** using the input parameters listed.

**Figure 3:**
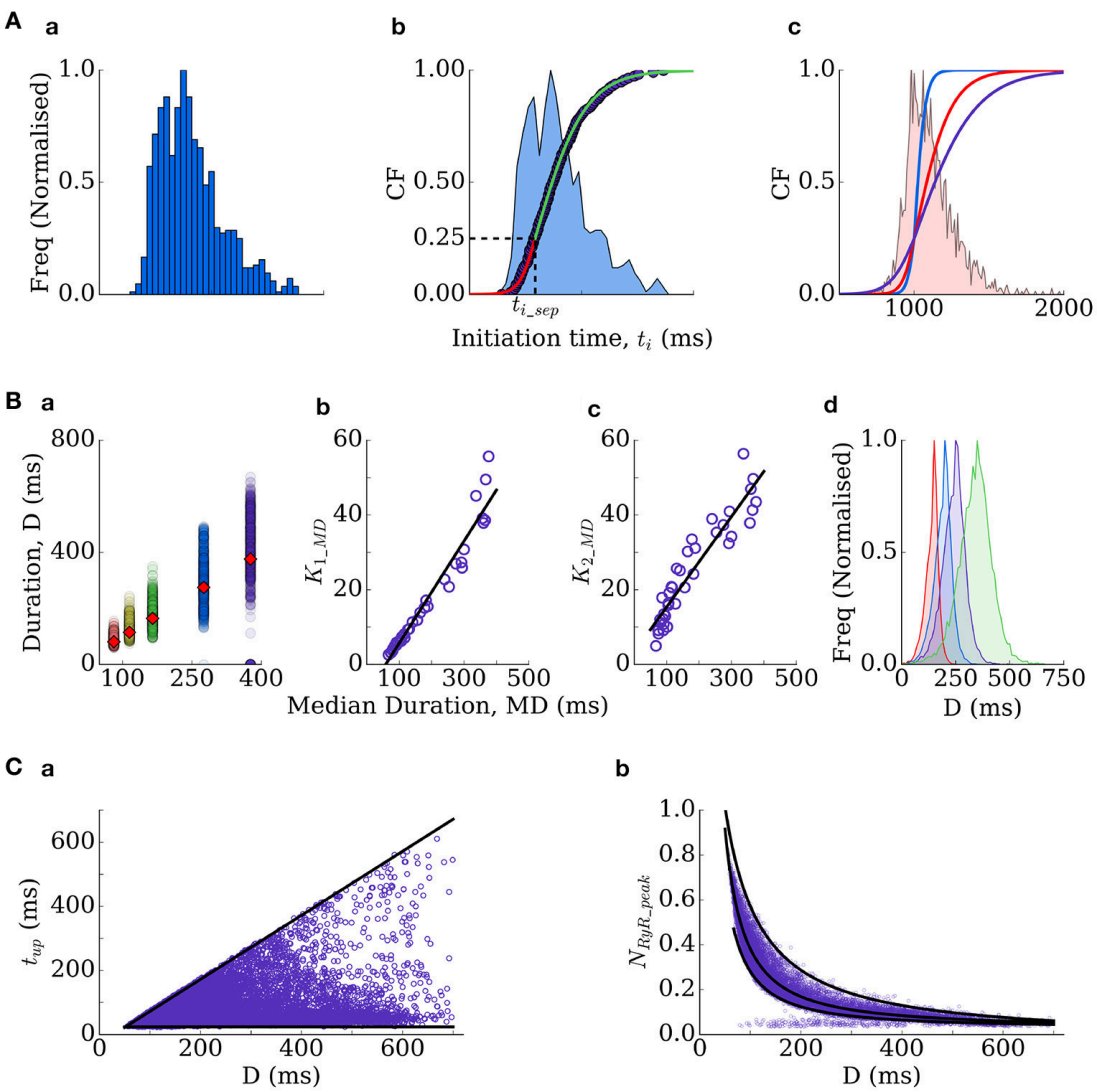
Derivation of the RyR waveform parameters. **(A)** Initiation time distributions: **(a)** is an example distribution produced by the spatial cell model; **(b)** is the cumulative frequency of the distribution (purple dots) and two sigmoidal functions (red and green lines) which approximate it–the distribution itself is shown for reference (blue shading); **(c)** cumulative frequencies of the three distributions used in this study (*t*_*i*_ distribution widths of 350 ms–blue; 550 ms–red; 1,000 ms–purple), with the histogram corresponding to the red distribution shown for reference. **(B)** Duration distributions: **(a)** scatter-plot of the durations associated with multiple simulations under different conditions (colors), plotted against the median of each distribution, which is also shown as the red diamonds for reference; **(b,c)** correlation of the gradient parameters of sigmoidal functions describing the distribution either side of the median with the median value; **(d)** four duration distributions used in the present study (Median Duration = 150 ms–red; 200 ms–blue; 250 ms–purple; 300 ms–green). **(C)** Other parameters correlate with the duration–time to peak (*t*_*up*_, **a**) and the waveform peak (*N*_*RyR*_*peak*_, **b**).

We therefore implemented a “non-spatial” simplification of the Ca^2+^ handling model (described in Isolated Cell Models–Intracellular Ca^2+^ Handling, above) for use in tissue-level simulations, to capture spontaneous Ca^2+^ release events at significantly reduced computational cost and with complete controllability. The non-spatial model consists of a single CRU (with RyR and LTCC dynamics solved deterministically) with additional analytical functions which describe the RyR waveform associated with whole-cell spontaneous release events, derived from analysis of the fully detailed spatial cell model, similar to the approach used in Campos et al. (2015) and Colman et al. (2015). For a simple transient-spike morphology (Figure 2) this function has the form:

$$\begin{array}{l} N_{RyR\text{\_}O}=\frac{N_{RyR\text{\_}peak}}{\left(1+e^{-\left(t-\left(t_{i}+0.5t_{up}\right)\right)/\left(0.1689t_{up}+0.00255\right)}\right)\left(1+e^{-\left(t-\left(t_{i}+t_{up}+0.5t_{decay}\right)\right)/\left(0.1689t_{decay}+0.00255\right)}\right)} \end{array}$$

where *t*_*i*_ is the initiation time of the spontaneous release and *t*_*up*_, *t*_*decay*_ and *N*_*RyR*_*peak*_ describe the shape of the waveform and are all determined from the *duration* (Figures 2, 3 and described below). The function for the plateau-like waveform (corresponding to durations longer than 250 ms) is derived from the same parameters (note the peak time, *t*_*p*_, is *t*_*i*_+*t*_*up*_):

$$\begin{array}{l} N_{RyR\text{\_}O}=\frac{N_{RyR\text{\_}plateau}}{\left(1+e^{-\left(t-\left(t_{i}+17.5\right)\right)/5.946}\right)\left(1+e^{\left(t-\left(t_{f}-17.5\right)\right)/5.946}\right)}\\ +\frac{N_{RyR\text{\_}peak}-N_{RyR\text{\_}plateau}}{\left(1+e^{-\left(t-\left(t_{p}-17.5\right)\right)/5.946}\right)\left(1+e^{\left(t-\left(t_{p}+17.5\right)\right)/5.946}\right)} \end{array}$$

Thus, the waveform is completely described by the *initiation time* and the *duration*. The stochastic nature of spontaneous Ca^2+^ release is captured by randomly selecting these parameters from functions describing their physiological distributions. The distributions describing initiation time of whole-cell spontaneous release events in the spatial cell model are typically skewed (Figure 3Aa) and well approximated by two sigmoidal functions, split around the cumulative probability of 0.25 (corresponding to a specific initiation time, *t*_*i*_*sep*_; Figure 3Ab). The initiation time can therefore be determined by passing a random number into the inverse of the two sigmoidal functions:

$$\begin{array}{l} t_{i}=\{\begin{array}{c} -k_{F1}\text{ln }\left(\frac{0.5}{rand}-1\right)+t_{i_{sep}},\;rand<0.25\\ -k_{F2}\text{ln }\left(\frac{1.5}{rand+0.5}-1\right)+t_{i_{sep}},\;rand\geq 0.25 \end{array} \end{array}$$

where the gradient parameters of the two sigmoidal functions (*k*_*F*1_, *k*_*F*2_) and the *t*_*i*_ at the cumulative probability of 0.25, *t*_*i*_*sep*_, completely control the resulting distribution. The duration, *D*, of the RyR waveform can be determined from distributions in an analogous manner:

$$\begin{array}{l} D=\{\begin{array}{c} k_{1\_MD}\text{ln }\left[1/\left(rand-1\right)\right]+MD,\;rand<0.5\\ k_{2\_MD}\text{ln }\left[1/\left(rand-1\right)\right]+MD,\;rand\geq 0.5 \end{array} \end{array}$$

where *MD* refers to the median and *k*_1_*MD*_ and *k*_2_*MD*_ are functions of the median (in conditions where longer waveforms are observed, the variability in waveform duration between simulations is larger; Figure 3Ba–c):

$$\begin{array}{l} k_{1\text{\_}MD}=0.1366MD-7.98\\ k_{2\text{\_}MD}=0.12MD-3.265 \end{array}$$

Thus, the distribution is entirely described by the median. Finally, *t*_*up*_ and *N*_*RyR*_*peak*_ can be determined from the given duration (Figure 3C):

$$\begin{array}{l} \;t_{up}=24+rand(D-52)\\ N_{RyR\_peak}=\{\begin{array}{c} MRYR-rand(159.59(D^{-1.327}-D^{-1.4})),\;rand<0.5\\ MRYR-(1-rand)(159.59\left({\left(D+30\right)}^{-1.15}+D^{-1.327}\right)+0.08),\;rand\geq 0.5 \end{array} \end{array}$$

Where *MRYR* refers to the median and is given by:

$$\begin{array}{l} MRYR=159.59D^{-1.327}+0.028 \end{array}$$

And if duration > 250 ms, it is also necessary to compute *N*_*RyR*_*plateau*_:

$$\begin{array}{l} N_{RyR\text{\_}plateau}=31.09{\left(0.01D\right)}^{-7.39}\\ \;+\left(rand-0.5\right)\left(-5\times 10^{-4}D\text{\_}0.0275\right)+0.34 \end{array}$$

These formulations therefore allow complete control over spontaneous release dynamics through just four parameters (*k*_*F*1_, *k*_*F*2_*, t*_*i*_*sep*_, median duration). We fix *t*_*i*_*sep*_ in simulations such that only the width of the initiation time distribution (*k*_*F*1_, *k*_*F*2_) and the median of the duration distribution are varied: *t*_*i*_ distribution widths of 350, 550, and 1,000 ms (Figure 3Ac) were used in tissue simulations; duration medians of 150, 200, 250, 300, and 350 ms (Figure 3Bd) were used in single-cell and tissue simulations. Derivation of these equations from the fully detailed spatial cell model ensures self-consistency and physiological validity of the resulting waveforms.

### Computational aspects

Models were coded in C/C++ and run on a Linux desktop machine, or using the University of Leeds ARC2 High Performance Computing facilities. Equations for isolated cell models were solved using a forward Euler method with a time step of Δ*t* = 0.05 ms; ion channel gating equations were solved using the Rush-Larsen scheme (Rush and Larsen, 1978). For tissue models, the monodomain equation was solved using a Forward Time Centred Space method: Space steps of Δ*x* = 0.2 mm were used in the 1D strand, Δ*x* = Δ*y* = 0.2 mm in the 2D tissue, and Δ*x* = Δ*y* = 0.425 mm and Δ*z* = 0.5 mm in the 3D wedge model (as defined by the diffusion tensor MRI dataset). Parallelisation was implemented with OpenMP. Cell APD was measured from the time the membrane potential crossed −80 mV during the upstroke of the action potential, to when the membrane potential crossed back over −80 mV during the repolarisation phase.

## Results

### The hERG activator MC-II-157c partially or fully reverses APD prolongation heterogeneously

**Figure 4:**
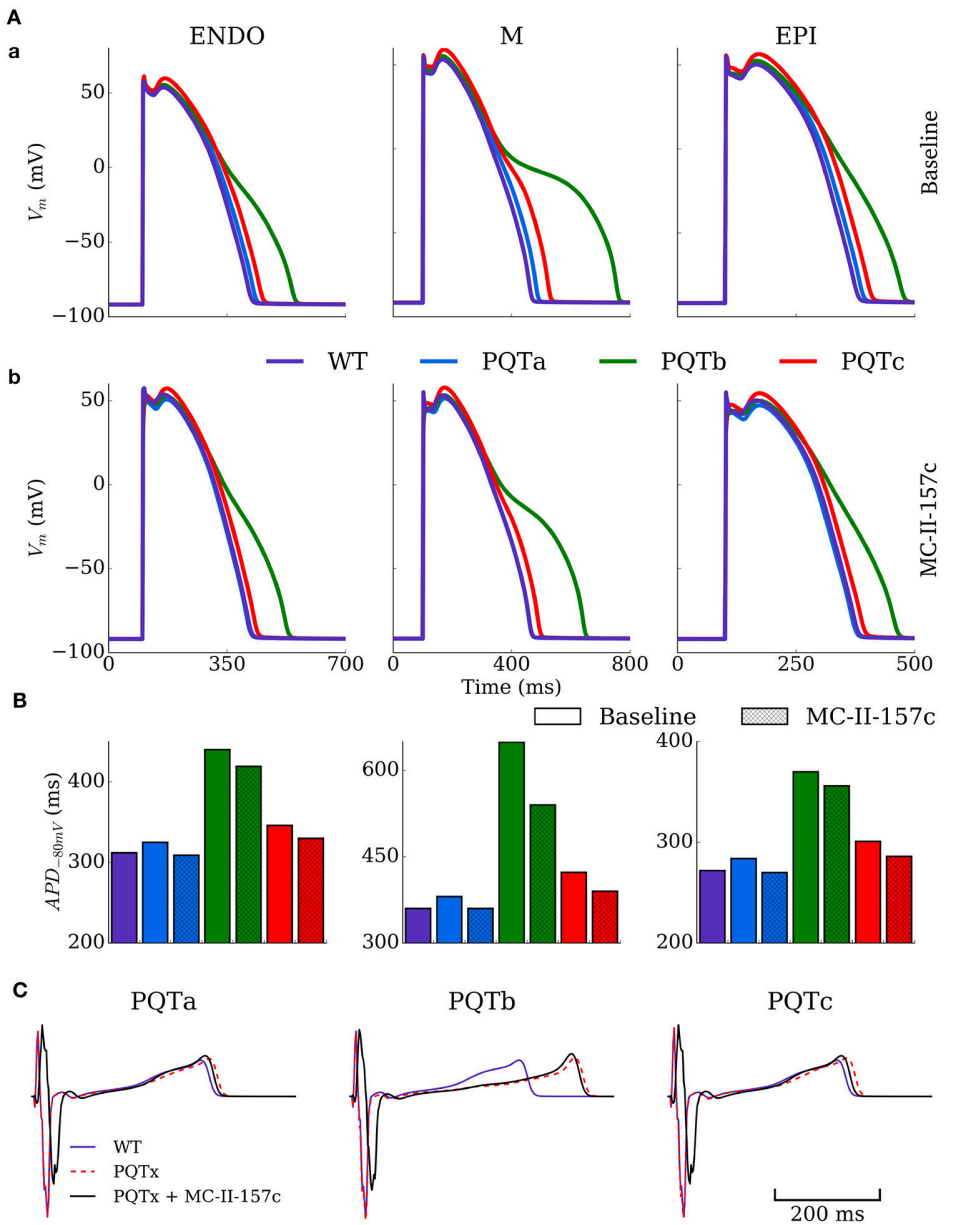
Action potential and ECG morphology and duration in PQT and MC-II-157c. **(A)** Action potentials for the three cell types (ENDO, left; M, middle; EPI, right) under baseline (upper) and MC-II-157c (lower) conditions in control (WT, purple) and PQTa-c conditions (blue, green, red) in the spatial cell model. **(B)** Action potential duration at −80 mV for the same conditions in **(A)**. The colors in the key apply to both panels **(A,B)**. **(C)** Computed ECGs for the three conditions (dotted red line) and with the application of MC-II-157c (black line), relative to the WT (purple line). MC-II-157c was modeled as *I*_Kr_ modification + 40% *I*_Na_ block. Any impact of *I*_Na_ block on the upstroke velocity is not clear at this scale.

The single cell model was paced at a cycle length of 1,000 ms for 100 beats under control (WT), PQT and PQT + MC-II-157c conditions to evaluate the efficacy of MC-II-157c on reversing PQT induced APD prolongation (Figure 4). Figure 4A shows effects on AP morphology of the PQTa, PQTb, and PQTc conditions in endocardial, midmyocardial, and epicardial cells, with WT action potentials shown as a reference. The PQTb condition (downregulation of *I*_Kr_) has the largest effect on prolonging APD (from 360 to 649 ms in midmyocardial cells, an increase of 80%), due to the larger *I*_Kr_ conductance in human cells relative to *I*_Ks_ conductance, and its primary role in AP repolarisation. All three PQT conditions increase the transmural difference in APD (the difference between the longest and shortest APDs in the three cell types), from 88 ms in WT to 97, 279 and 122 ms with PQTa, PQTb, and PQTc, respectively.

Figure 4B shows how the hERG activator MC-II-157c reduces APD back towards control levels in all cell types and with all PQT conditions. The drug reduced APD back to control levels in PQTa (downregulation of *I*_Ks_) due to the minimal effect this condition has on initially prolonging APD, but the largest decrease was seen with PQTb in midmyocardial cells, where APD was reduced from 649 to 540 ms, a reduction of 17% (Figure 4C). It should be noted, however, that MC-II-157c has transmurally heterogeneous effects; that is, the degree of APD reduction seen in the three different cell types is not identical, with the drug under PQTb conditions (for example) giving a 5% decrease in endocardial cells, 17% in midmyocardial cells and 4% in epicardial cells. Consequently, the maximal transmural difference in APD in PQTb reduces from 279 ms with the PQT condition alone to 184 ms with PQTb plus MC-II-157c, but does not reduce transmural difference in APD back down to WT levels (184 vs. 88 ms).

ECGs were computed for the different conditions using the 3D ventricular wedge model under normal pacing (BCL = 1,000 ms). QT-prolongation was observed for all three remodeling types (Figure 4C), with PQTb exhibiting the longest QT-interval, congruent with single cell results (QT = 310 ms in WT compared to 326, 418, and 329 ms in PQTa-c, respectively). MC-II-157c resulted in a delay in the QRS peak as well as earlier absolute repolarisation time and consequent shortening of the QT interval (QT = 309 ms, 403 and 310 ms in PQTa-c + MC-II-157c; Figure 4C); in PQTa and PQTc, MC-II-157c fully reverses QT prolongation.

**Figure 5:**
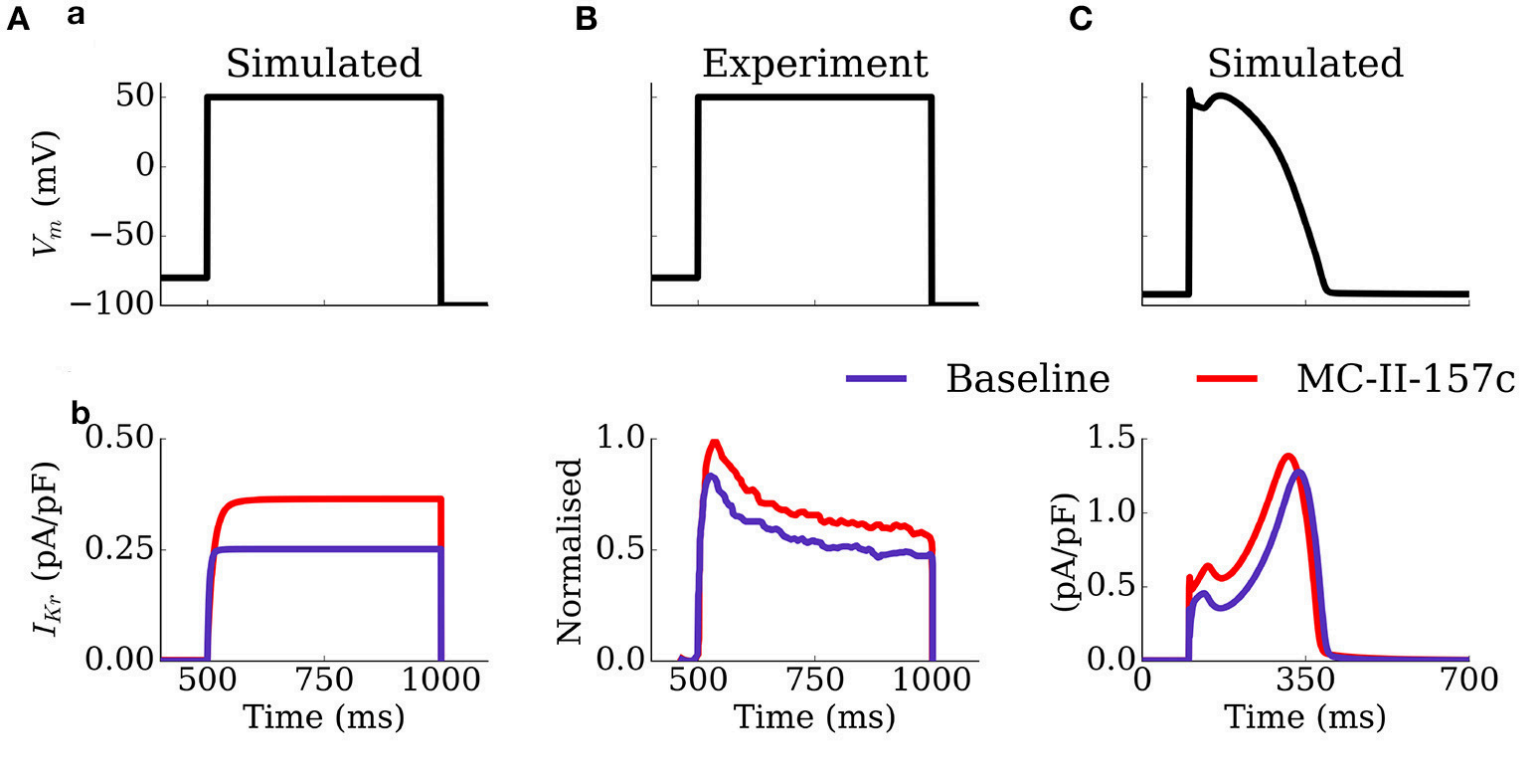
Activity of *I*_Kr_ during voltage and AP clamp conditions under the application of MC-II-157c. *I*_Kr_ traces **(b)** elicited by a voltage step protocol **(a)** in simulation **(A)** and experiments of Guo et al. (2014); **(B)** and elicited by a simulated AP clamp **(C)**, for baseline (purple) and MC-II-157c (red) conditions. In **(B)**, the current traces have been normalized to the peak of the control current. Conditions in **(A)** were matched to those experimentally—the intracellular and extracellular concentrations of potassium were set to 120 and 5.4 mM, respectively.

The mechanism by which MC-II-157c shortens APD, despire reducing the maximal conductance of *I*_Kr_, is illustrated in Figure 5: the shifts in the activation and inactivation curves and the slowing of deactivation kinetics result in an increased current during both voltage clamp and AP clamp experiments. These results are generally congruent with the original study of Guo et al. (2014), although the current traces do differ in morphology and extent of effect of MC-II-157c (see Limitations).

### MC-II-157c has PQT-type dependent effectiveness in reversing SR loading and spontaneous release events

**Figure 6:**
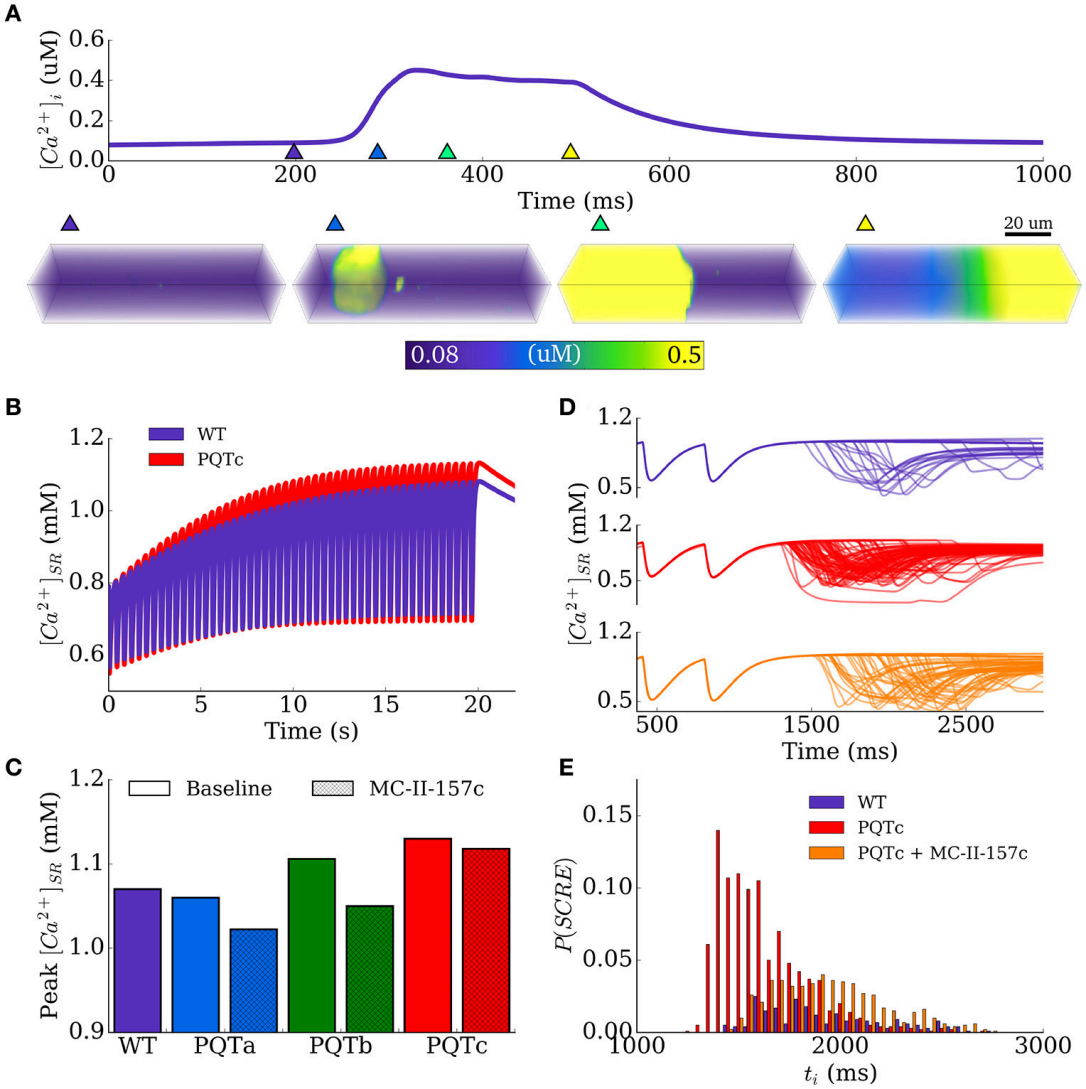
SR Ca^2+^ loading and spontaneous Ca^2+^ release events. **(A)** Example of a whole-cell spontaneous Ca^2+^ release event (Ca^2+^-wave), using the spatial model of stochastic intracellular Ca^2+^ handling, showing the Ca^2+^ transient (upper) and snapshots of Ca^2+^ concentration in the 3D cell volume (lower). The triangular makers in the upper panel indicate the timings of the snapshots in the lower panel. **(B)** Time-series of SR-Ca^2+^ during the SR-loading protocol, shown for control (WT, purple) and PQTc (red). **(C)** Peak SR-Ca^2+^ during the loading protocol for all conditions. **(D)** Examples of spontaneous release resulting from SR-loading for control (WT, purple), PQTc (red) and PQTc + MC-II-157c (orange). 100 simulations of each conditions are shown to indicate variation in spontaneous release. **(E)** Distributions of initiation time of spontaneous release events, corresponding to the same conditions shown in **(D)**. The time is relative to the start of the simulation (3 beats, initial conditions of dynamic steady state) to align with **(D)**. MC-II-157c was modeled as *I*_Kr_ modification + 40% *I*_Na_ block.

The vulnerability to the emergence of whole-cell spontaneous Ca^2+^ release events (such as intracellular Ca^2+^ waves, Figure 6A) is primarily controlled by the dynamics of the intracellular Ca^2+^ handling system and the SR Ca^2+^ load, wherein cells typically exhibit an SR load threshold above which the probability of spontaneous release events significantly increases (Wagner et al., 2015). The focus of this study is not on the mechanisms of spontaneous Ca^2+^ release, and therefore investigation of Ca^2+^ handling remodeling is beyond its scope. However, APD prolongation associated with PQT, and its subsequent reversal by MC-II-157c, may influence SR loading and consequently the vulnerability to the emergence of whole-cell spontaneous Ca^2+^ release.

An SR-loading protocol was used to analyse this behavior, by pacing the spatial cell model at a rapid rate (cycle length of 400 ms). The maximal flux rate of intracellular uptake was increased, simulating the effect of sympathetic stimulation, in order to promote SR loading. An increase by a factor of two was chosen as this loaded the SR-Ca^2+^ in the WT model to just above the spontaneous release threshold; the most suitable region to reveal the consequence of APD modulation on spontaneous activity.

The time series of SR-Ca^2+^ in WT and the PQTc condition in isolated epicardial cells illustrates the effect on SR-Ca^2+^ loading under rapid pacing and highlights that APD prolongation associated with PQT can promote loading (Figure 6B). The peak of the SR Ca^2+^ concentration during this time provides a measure of SR Ca^2+^ loading; these data are shown in Figure 6C for WT and all PQT conditions with and without MC-II-157c. The PQTa condition (downregulation of *I*_Ks_) does not promote SR loading compared to WT (peak SR Ca^2+^ load of 1.07 mM in WT and 1.06 mM in PQTa), but the PQTb (downregulation of *I*_Kr_) and PQTc (upregulation of *I*_Ca,L_) conditions do (peak SR Ca^2+^ load of 1.106 and 1.13 mM, respectively), with PQTc having the largest effect due to the increase in the transmembrane Ca^2+^ current in this condition. MC-II-157c reverses SR Ca^2+^ loading in PQTb, reducing peak SR Ca^2+^ load at periodic steady-state to 1.05 mM, which is below WT levels. However, the drug has only a very small effect in PQTc (down to 1.108 mM) due to the minimal effect MC-II-157c has on APD in this condition, and the subsequently small change in the time course of *I*_Ca,L_.

These relatively small changes in SR-Ca^2+^ can manifest as significant differences in the vulnerability to spontaneous Ca^2+^ release due to the non-linear threshold dependence on SR Ca^2+^ concentration (Figures 6D,E). The probability of whole-cell spontaneous release events and the probability distributions describing the initiation time can be computed from a large set of simulations (*N* = 1,000 per condition). Example distributions are shown in Figure 6E for the WT and PQTc with and without MC-II-157c, highlighting that even the small change in SR Ca^2+^ as a result of MC-II-157c significantly reduces the probability of spontaneous release (~50% in PQTc with MC-II-157c compared to 98% in PQTc alone) as well as widening the distribution (although not fully reversed to WT). Due to the choice of loading parameters giving the WT close to threshold, no spontaneous release occurs for either PQTa or PQTb under the application of MC-II-157c as in these conditions threshold SR Ca^2+^ is not reached.

### MC-II-157c is effective in inhibiting DADs turning into triggered activity

The effect of MC-II-157c on the probability of DADs manifesting as full triggered action potentials was investigated using the simplified, non-spatial model such that spontaneous release waveforms could be directly controlled. The initiation time was set to 1,000 ms and behavior of the cell models for WT, PQT and PQT + MC-II-157c was compared over multiple simulations (*N* = 1,000 per condition) for four different RyR waveform duration distributions (see details in Simulating Spontaneous Release Events in Tissue Models; the distribution determines the values of duration which can be selected from a random number input and thus the actual value of the duration will vary randomly within the 1,000 simulations according to the given distribution).

**Figure 7:**
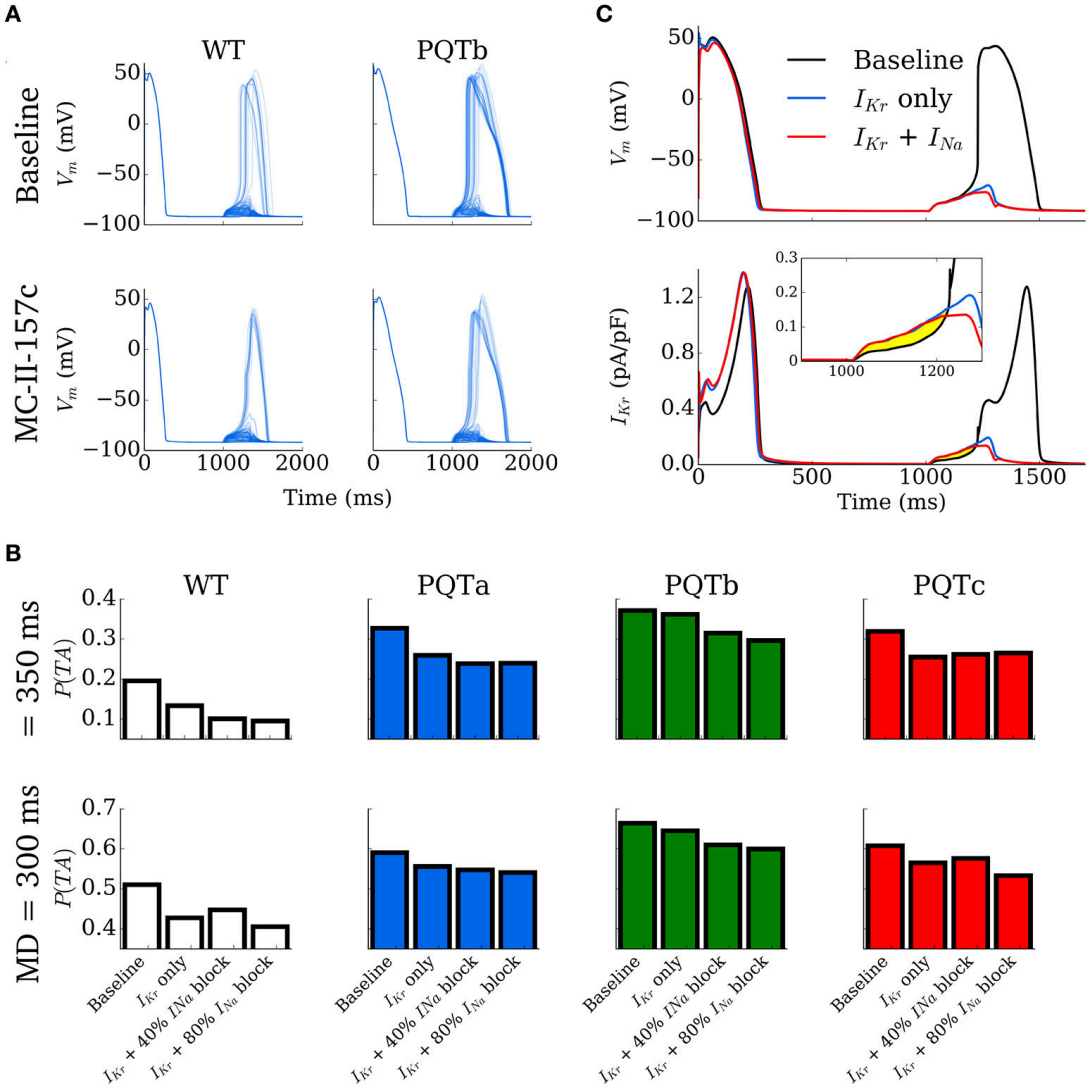
Development of triggered action potentials in single cell. **(A)** 100 Action Potential traces for control (WT, left) and PQTb (right) under baseline (upper) and MC-II-157c (lower) conditions, associated with spontaneous Ca^2+^ release events. A proportion of the DADs manifest as triggered action potentials in all conditions. **(B)** Probability of DADs triggering full action potentials for control (WT) and PQTa-c under baseline and MC-II-157c conditions. The degree of *I*_Na_ block associated with MC-II-157c is varied between 0% (*I*_Kr_ only), 40 and 80%. The upper panel corresponds to a median RyR waveform duration of 350 ms; the lower panel to 300 ms. **(C)** Illustration of the mechanism by which MC-II-157c inhibits triggered activity, showing that the larger *I*_Kr_ (lower panel and inset) acts to oppose depolarising currents during the DAD. Yellow highlighted region clearly illustrates the differences between the conditions.

Examples of 100 simulations for WT and PQTb, with and without MC-II-157c, are shown in Figure 7A. In PQTb, more DADs turn into triggered activity compared to in WT (top two panels), while simulated application of MC-II-157c reduces the occurances of triggered activity (bottom two panels). These data are summarized for all conditions and two duration distributions (median 300 and 350 ms, see Simulating Spontaneous Release Events in Tissue Models) in Figure 7B, wherein the degree of block of *I*_Na_ associated with MC-II-157c is varied (0, 40, and 80% block). In all cases, the *I*_Kr_ modification reduces the probability of triggered activity (defined as the number of simulations in which triggered activity occurred as a proportion of the total), but the role of *I*_Na_ is less clear; blocking *I*_Na_ is important, but the degree of *I*_Na_ block has different effects depending on the condition. The mechanism of the drug's action is shown in Figure 7C: principally, an increase in repolarising *I*_Kr_ acts during the DAD to keep the cell's membrane potential below the threshold for triggered activity; reduced *I*_Na_ also pays a role (although not as great as that of *I*_Kr_) in the drug's mechanism of action as it reduces excitability of the cell, again reducing the ease with which the cell's membrane potential can reach threshold.

### MC-II-157c reduces vulnerability to ectopic activity in tissue

The vulnerability to the development of ectopic activity (i.e., propagation of triggered activity in all directions through the tissue) was assessed in a 2D homogeneous sheet to provide a medium for the synchronization of independent stochastic release events. Ten simulations were performed for each condition (WT and PQT variants ± MC-II-157c for the combinations of *t*_*i*_ and duration median distributions; Epicardial cell model) wherein the tissue model was paced to steady state and then left quiescent for two simulation seconds within which time the spontaneous release occurs.

The occurrence of ectopic activity exhibits a largely “all-or-nothing” response, where most conditions lead to either 0 or 100% of simulations resulting in a premature excitation (Table 1). Ectopic activity was promoted by narrow distributions of initiation time (i.e., tight synchronization) and short RyR waveforms (i.e., large spontaneous Ca^2+^ transients), and conversely inhibited by wide distributions of initiation time (i.e., lose synchronization) and long RyR waveforms (i.e., small spontaneous Ca^2+^ transients). For example, no ectopic activity was observed for any condition with *t*_*i*_ distribution widths of 1,000 ms or duration medians of 300 ms or longer, whereas a *t*_*i*_ distribution width of 350 ms combined with median durations of 150 or 200 ms resulted in ectopic activity occurring in 100% of simulations (Table 1).

**Table 1:** Incidence of ectopic activity in 2D tissue under WT and PQT ± MC-II-157c conditions.

***t_*i*_* width**	**1,000**				**550**				**350**			
**Duration median**	**300**	**250**	**200**	**150**	**300**	**250**	**200**	**150**	**300**	**250**	**200**	**150**
WT	0	0	0	0	0	0	0	0	0	0	10	10
PQTa	0	0	0	0	0	0	0	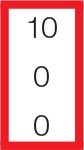	0	0	10	10
PQTa+C	0	0	0	0	0	0	0		0	0	10	10
PQTa+C_Na_	0	0	0	0	0	0	0		0	0	10	10
PQTb	0	0	0	0	0	0	10	10	0	10	10	10
PQTb+C	0	0	0	0	0	0	10	10	0	10	10	10
PQTb+C_Na_	0	0	0	0	0	0	10	10	0	10	10	10
PQTc	0	0	0	0	0	0	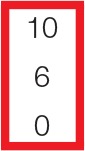	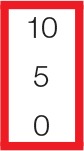	0	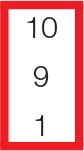	10	10
PQTc+C	0	0	0	0	0	0			0		10	10
PQTc+C_Na_	0	0	0	0	0	0			0		10	10

*The number of simulations where a premature excitation was observed is shown for each condition (out of a total of 10). The compound MC-II-157c is denoted by a “C” with I_Kr_ modification alone and “C_Na_” with inclusion of 40% block of I_Na_. Highlighted with red borders are the conditions in which MC-II-157c inhibits the development of ectopic activity*.

PQT variants were more susceptible to the development of ectopic activity in tissue than WT, congruent with the single-cell results (See MC-II-157c Is Effective in Inhibiting DADs Turning into Triggered Activity). Similarly in-line with single-cell results, MC-II-157c can inhibit ectopic activity (Table 1): for example, it reduces or entirely inhibits the occurrence of premature excitation in four conditions: PQTa with *t*_*i*_ width of 550 ms and duration median 150 ms; PQTc with *t*_*i*_ width of 550 ms and duration median 200 ms; PQTc with *t*_*i*_ width of 550 ms and duration median 150 ms; and PQTc with *t*_*i*_ width of 350 ms and duration median 250 ms. The effect of *I*_Na_ block is also congruent with single cell results: it contributes to the inhibition of ectopic activity, but to a smaller extent than *I*_Kr_ modification.

**Figure 8:**
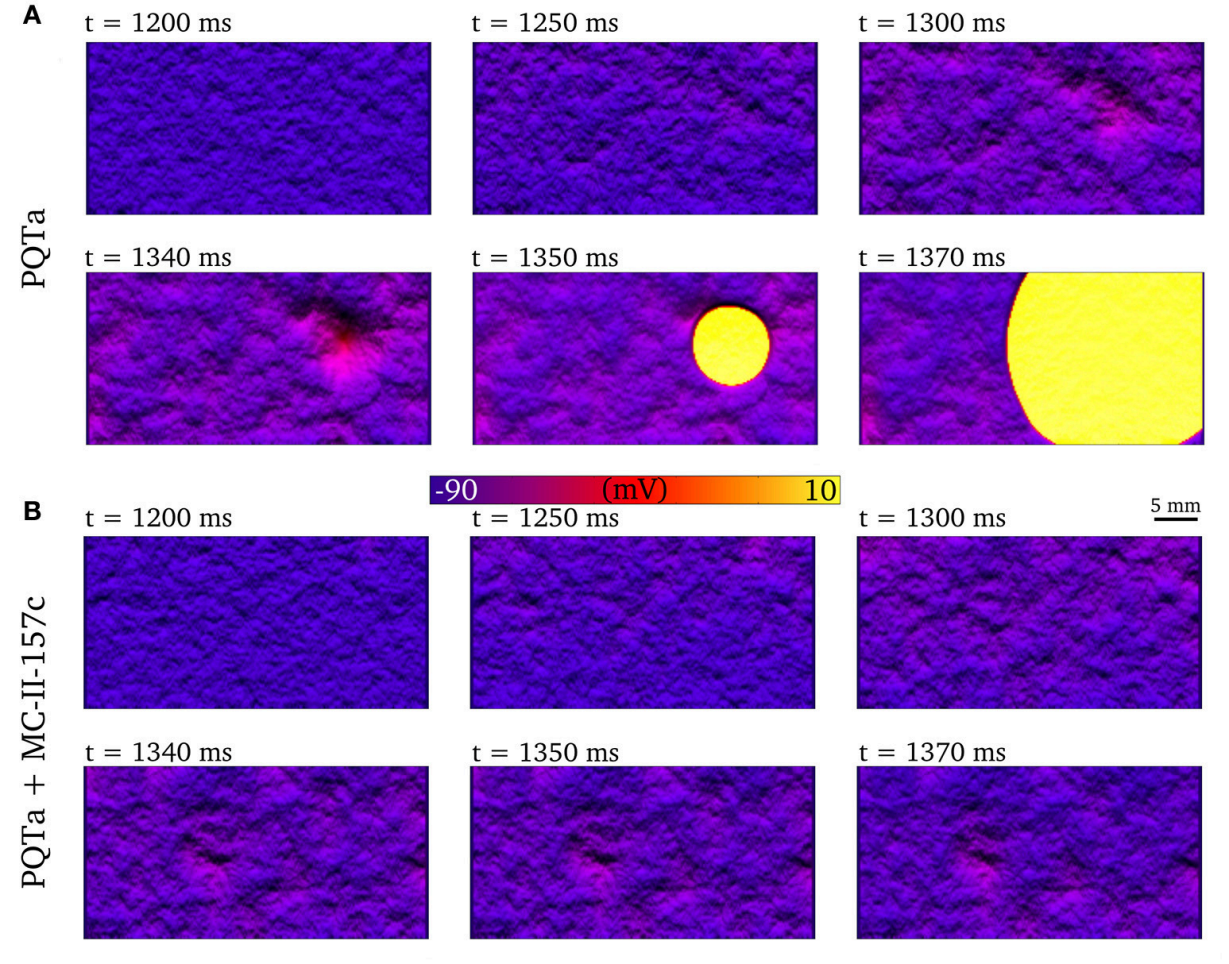
Development of premature excitation in 2D tissue sheets. Temporal snapshots of the membrane voltage in a 2D slice of tissue during spontaneous Ca^2+^ release events for PQTa **(A)** and PQTa + MC-II-157c (*I*_Kr_ modification + 40% *I*_Na_ block, **(B)**. The color bar has been scaled to emphasize voltage differences in the DAD region and lighting has been added to further enhance local variation in voltage. Data corresponds to *t*_*i*_ width of 550 ms and duration median 150 ms.

Figure 8A shows an example of synchronized triggered activity initiating in a region of the 2D tissue (shown by the clustered peaks the images), before this triggered activity spreads throughout the tissue (shown by the yellow region) as an ectopic propagation. When the same situation is simulated with MC-II-157c (Figure 8B), triggered activity, and therefore ectopic propagation, is inhibited.

### MC-II-157c increases the vulnerable window in tissue

An S1-S2 pacing protocol was applied to the 1D strand model in order to compute the vulnerability window: S2 stimuli were applied across a range of time intervals, centered on one in every five cells of the 100 comprising the model, from the tenth to the 90th.

**Figure 9:**
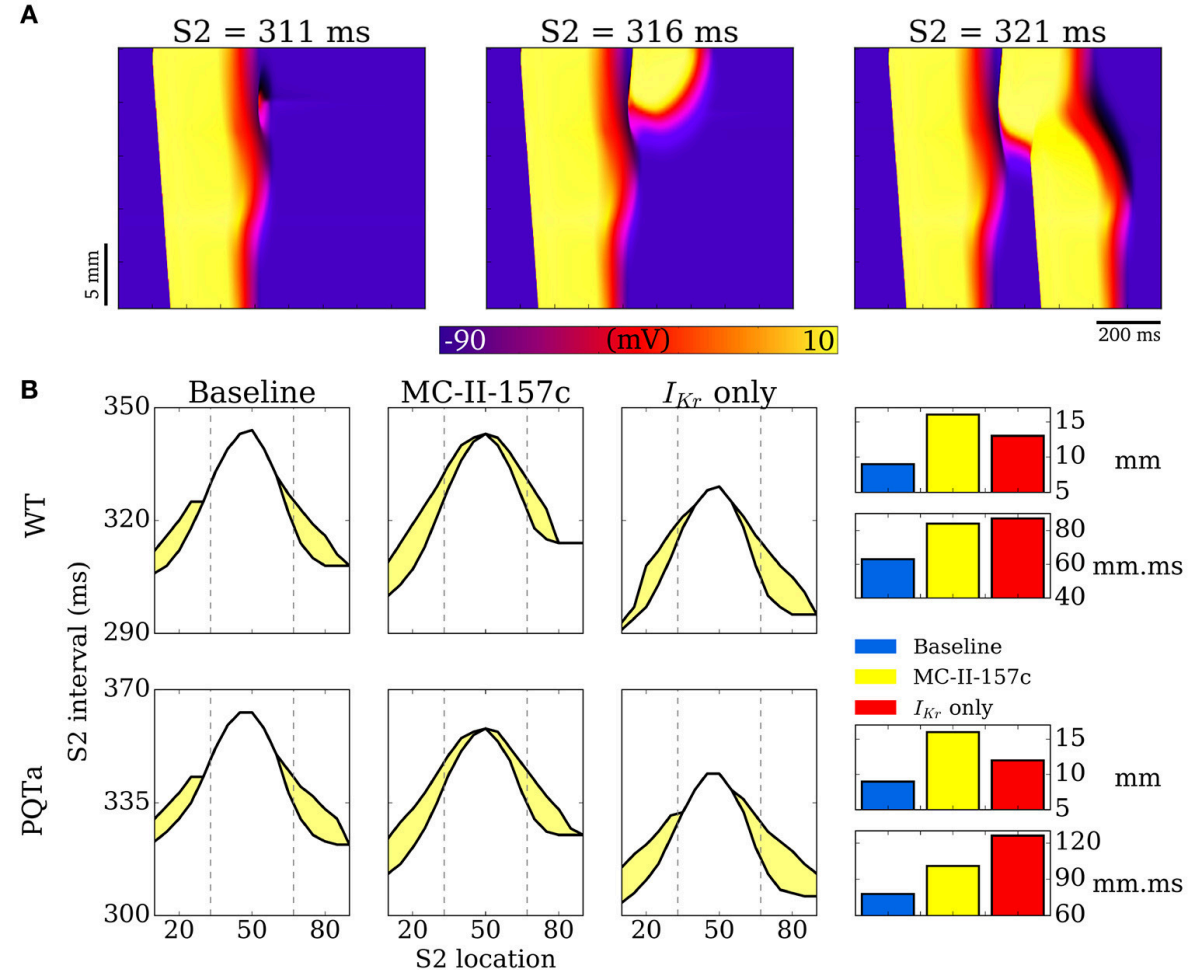
Vulnerability windows to unidirectional conduction block. **(A)** Examples of propagation following applied S2 stimulus at different time intervals, illustrating conduction block (left), unidirectional conduction (middle) and full conduction (right). **(B)** Vulnerability windows for control (WT, upper) and PQTa (lower) under baseline, MC-II-157c (*I*_Kr_ modification + 40% *I*_Na_ block) and *I*_Kr_ modulation alone conditions. The lower line in each plot represents the refractory period (time below which no propagation occurs); the upper line represents the earliest time of bidirectional conduction; the area between the lines (shaded yellow) is the region within which unidirectional conduction occurs. Right panel is bar charts summarizing the length (mm) and area (mm.ms) of the vulnerability windows. Dotted lines illustrate the regions of the different cell types (ENDO, left; EPI, right).

Examples of propagation applied during the repolarisation phase in 1D tissue simulations are shown in Figure 9A: if the triggered activity occurs early (at 311 ms in this example), the resultant excitation is surrounded by refractory tissue and the triggered activity dies out without propagating; If the triggered activity occurs slightly later (e.g., 316 ms) then refractory tissue is encountered at only one side of the triggered activity site and unidirectional block (or unidirectional propagation) occurs, in the retrograde direction (back toward the endocardium) in this example; If triggered activity occurs later than this (321 ms in this example) then all surrounding tissue has recovered and the triggered activity propagates in both directions along the strand (i.e., ectopic propagation, analogous to the situation shown in 2D tissue in Figure 8A). It is the unidirectional block situation (i.e., when the triggered activity occurs in the VW) that can lead to re-entrant arrhythmias if this situation occurred in 2D or 3D tissue. The VW identifies occurrences of trigger and substrate interaction that may lead to arrhythmias, and so quantifying the size VW is a convenient method to examine effects of disease conditions and drugs on trigger and substrate interaction. The baseline VWs for WT and the PQTa condition are mapped out in Figure 9B, as well as VWs in these two situations with simulated addition of MC-II-157c (*I*_Kr_ modification plus 50% *I*_Na_ block), as well as with only the MC-II-157c *I*_Kr_ modification. These VWs are quantified by length (over which they occur in the 1D strand) and area (length × temporal width). In both WT and PQTa conditions, the drug increases both the length and the area of the VW (for example, length increases from 9 to 16 mm in the PQTa condition, and area increases from 78 to 101 mm.ms). Again, the influence of *I*_Na_ is different depending on the condition: including the effects of *I*_Na_ (i.e., the full MC-II-157c simulations compared to the *I*_Kr_ only simulations) increases the length of tissue over which the VW occurs in all conditions (e.g., from 12 to 16 mm in PQTa), but reduces the overall area of the VW (e.g., from 126 to 101 mm.ms).

## Discussion

We used a multi-scale computational modeling approach to examine the interactions between cardiac arrhythmia trigger and substrate in general conditions associated with prolonged QT intervals, and their modulation by the hERG activator and sodium channel blocker MC-II-157c. Although we examined the effects of pharmacological modification on trigger and substrate interaction using a specific hERG activator, and gained novel insights into how modification of the depolarising (*I*_Na_) and repolarising (*I*_Kr_) membrane ionic currents targeted by the drug affects arrhythmia triggers and substrates, our findings also provide general insight into the role of ion-currents in controlling triggers and substrate at multiple scales which, along with insight from other *in silico* studies [see (Dutta et al., 2016; Mann et al., 2016) for recent examples], may be applicable to other pharmacological compounds that modify membrane ionic currents as well as pro-arrhythmic electrical remodeling.

### Key findings

Our key findings are that: (i) Despite the hERG activator MC-II-157c reducing the maximal conductance of *I*_Kr_ by 12% compared to WT, the drug's modifications to *I*_Kr_ activation, inactivation and deactivation kinetics result in an overall increase in *I*_Kr_ during the action potential, and a concomitantly reduced APD under all PQT conditions in all transmural cell types; (ii) Although MC-II-157c acts on membrane ionic currents carrying K^+^ and Na^+^, the drug has indirect effects on intracellular Ca^2+^ handling, particularly SR Ca^2+^ loading and related spontaneous SR Ca^2+^ release events and subsequent DADs, through modulation of the AP; (iii) Increased *I*_Kr_ (through its repolarising effects) and reduced *I*_Na_ (by decreasing cell excitability) act to reduce the probability of a DAD reaching threshold and developing into triggered activity at both cellular and tissue scales; and (iv) Despite MC-II-157c reducing triggered activity at the cell level, the drug can increase both the spatial region of tissue over which a VW for unidirectional conduction block occurs, as well as the temporal width of the VW at all points along the tissue, and in doing so increases the total spatiotemporal size of the VW. These results highlight the complex considerations which underlie overall vulnerability to arrhythmia at multiple scales.

### Efficacy of MC-II-157c as an anti-arrhythmic drug

At the isolated cell level, MC-II-157c reduces SR Ca^2+^ loading, reduces the occurrences of spontaneous SR Ca^2+^ release events, reduces DAD occurrences, and reduces the probability of a DAD developing into a triggered action potential. At the tissue level, the combination of these factors leads to a significantly reduced vulnerability to the development of ectopic beats; MC-II-157c reduces the probability of an ectopic beat development at given spontaneous release function distributions (i.e., synchronization degree) as well as inhibiting SR Ca^2+^ loading and thus reducing synchronization (which, itself, reduces the probability of ectopic beats). In relation to the development of Ca^2+^ induced triggers at single cell and tissue levels, therefore, our simulation results suggest that the drug has efficacy as an anti-arrhythmic.

However, even though arrhythmic triggered activity is reduced with the drug, the spatiotemporal area of the VW increased, and so the anti-arrhythmic effects of the reduced probability of an arrhythmia trigger occurring is opposed by the pro-arrhythmic effects of an increased arrhythmia substrate. This highlights the importance of examining the effects of pharmacological compounds on both the triggers *and* the substrates that underlie arrhythmia initiation: there is a delicate balance between increased/decreased trigger and increased/decreased substrate (i.e., trigger-substrate interaction) that determines whether any given trigger stimulus (e.g., a spontaneous SR Ca^2+^ release event) will result in unidirectional propagation and, potentially, initiation of a re-entrant arrhythmia. This is one potential reason that single cell studies showing efficacious effects of putative anti-arrhythmic drugs may not translate to the clinic.

The effects of MC-II-157c on the VW occur through two mechanisms due to the drug's action on both *I*_Kr_ and *I*_Na_, both of which modify transmural dispersion of repolarisation. Activation of *I*_Kr_ causes heterogeneous changes to APD at the tissue level, the same mechanism as in our previous studies examining the effects of NS1643 (Peitersen et al., 2008). Block of *I*_Na_ results in slowed transmural conduction (Kléber and Rudy, 2004) and therefore delayed activation of epicardial (but not endocardial) tissue: this in turn modifies transmural dispersion of repolarisation, even though the change to APD is minimal with *I*_Na_ block. Block of *I*_Na_ also reduces excitability of the tissue, necessitating a larger trigger to initiate propagation, which also contributes to the change in the VW.

### Varying effects of sodium current block

The role of *I*_Na_ loss of function in arrhythmogenesis has been examined in detail previously (see Clancy et al., 2015 for a review); however, one intriguing finding from our cell and tissue simulations was the varying effects that different magnitudes of *I*_Na_ block had on trigger development and substrate size. At the cell level, the probability of triggered activity developing from DADs did not follow a simple monotonic change with increasing *I*_Na_ block in all cases. Take, for example, the 350 ms Ca^2+^ release duration distribution results shown in the top panels of Figure 7B: In WT and the PQTb condition, *I*_Na_ block (in addition to the *I*_Kr_ modification) reduces the probability of DADs developing into triggered activity (relative to the *I*_Kr_ modification alone), with more block reducing this probability; that is to say, the greater the *I*_Na_ block, the more anti-arrhythmic (in terms of reducing triggered activity) the effects. However, in the PQTa condition, while 40% block of *I*_Na_ reduced the probability of triggered activity occurring, increasing block of the current to 80% slightly increased the probability of triggered activity; in this condition, a small amount of *I*_Na_ block has anti-arrhythmic effects, but increasing this small level of block is pro-arrhythmic. In the PQTc condition, *I*_Na_ block of any magnitude increased the probability of triggered activity (i.e., *I*_Na_ block is pro-arrhythmic), with the probability of triggered activity occurring increasing as *I*_Na_ block is increased. Similarly varied results were found for the 300 ms Ca^2+^ release duration distribution (lower panels in Figure 7B), although the anti-/pro-arrhythmic effects did not necessarily match those seen with the 350 ms distribution.

One further note of caution with regards to *I*_Na_ block comes from our tissue-level VW results in Figure 9B. Quantification of the length of tissue over which the VW occurs shows that 50% *I*_Na_ block (compare the full MC-II-157c effects to the effects with the *I*_Kr_ modification alone) increases this length, potentially due to an increase in transmural dispersion of repolarisation with *I*_Na_ block; analyses of these results alone would conclude that *I*_Na_ block is pro-arrhythmic. However, despite the spatial width of the VW increasing, block of *I*_Na_ results in the total spatiotemporal area of the VW decreasing (again, compare the full MC-II-157c effects to the effects with the *I*_Kr_ modification alone), i.e., an anti-arrhythmic result, likely due to the reduced excitability that results from *I*_Na_ block reducing the likelihood that any triggered activity would propagate.

Our tissue-level findings therefore indicate that *I*_Na_ block *per se* is an effective antiarrhythmic strategy (as seen with class I antiarrhythmic drugs; Camm, 2012), but our cell-level findings highlight that the magnitude of *I*_Na_ determines cell (and by extension, tissue) electrophysiological consequences in a manner that is not intuitive. The mechanisms underlying these varying effects of *I*_Na_ block on arrhythmogenesis, particularly on DAD initiation and their development into triggered activity, remain to be elucidated.

### Modifying abnormal intracellular calcium handling through membrane current modification

Although this study did not focus on the mechanisms of arrhythmia triggers (in that we prescribed SR Ca^2+^ release events under certain conditions), one interesting finding did emerge in relation to arrhythmia triggers that can result from abnormal intracellular Ca^2+^ handling: Modification of membrane ion channels carrying ions other than Ca^2+^ (K^+^ and Na^+^ in this case) can have beneficial effects in terms of restoring abnormal intracellular Ca^2+^ handling, through their actions in shortening APD. This was shown in Figure 6, where the increased SR Ca^2+^ loading (and resultant spontaneous SR Ca^2+^ release events) seen under PQT conditions was reversed by upregulating *I*_Kr_ and downregulating *I*_Na_, which in turn shortened APD. This reduces the duration over which *I*_Ca,L_ is activated, reduces the amount of Ca^2+^ crossing the cell membrane and entering the cell via that current, and thus reduces SR Ca^2+^ load. Furthermore, the combined action of both of these current modifications reduced the probability of DADs manifesting as triggered action potentials in single cell as well as triggered action potentials manifesting as fully propagating ectopic beats at the tissue scale. Targeting cell membrane ion channels carrying ions other than Ca^2+^ in order to restore abnormal intracellular Ca^2+^ handling (as seen in heart failure, for example; Lou et al., 2012) may be beneficial in cases where up/downregulation of Ca^2+^-specific drug targets (ryanodine receptors or SERCA, for example) will alter the delicate homeostasis of an already-compromised system, yielding negative results (Ratner, 2015).

### Development of arrhythmic conduction patterns

**Figure 10:**
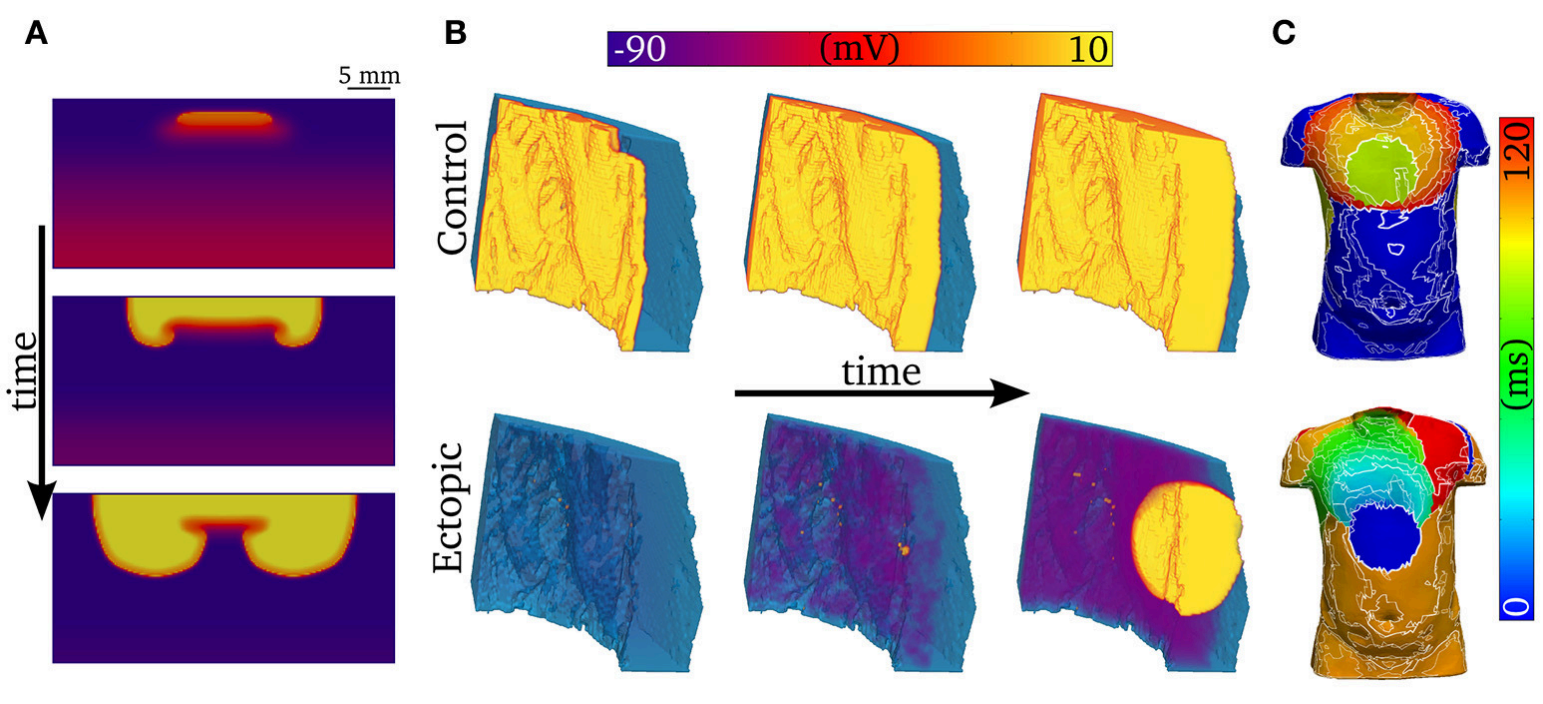
Examples of abnormal electrical excitation. **(A)** Example of conduction block in a 2D sheet, analogous to that observed in the 1D strand for computation of the vulnerability windows. Three snapshots in time are shown to illustrate the potential of unidirectional conduction block to develop into asymmetric, re-entrant-like activity. **(B)** Propagation patterns in the 3D ventricular wedge, showing normal, control pacing (upper panel) and a spontaneous ectopic beat (lower panel). The propagation of DADs, preceding the ectopic beat, is clear by the purple colored regions in the lower center panel. The voltage color bar corresponds to both panels **(A,B)**. **(C)** Body-surface potential activation maps (time at which potential > 0.1 mV) associated with normal and ectopic propagation, highlighting the significant difference in spatial patterns between the two conditions.

One-dimensional models (other than 1D rings; e.g., Vinet and Roberge, 1994) cannot simulate re-entrant activity, and so it is necessary to use 2D and 3D models to examine how unidirectional propagation develops into re-entry. Although the quantitative characteristics of the VW examined in 1D tissues may change in 2D and 3D depending on the spatial locations of cell types (e.g., “base-apex” as well as transmural distributions), based on our previous work we would expect the qualitative characteristics to remain similar (Benson et al., 2007, 2008, 2011a). We show examples of 2D and 3D modeling in Figure 10: In Figure 10A, a trigger occurring in the VW (resulting in unidirectional conduction block) develops into re-entry in a simple 2D model; In Figure 10B, a trigger occurring outside the VW develops into ectopic propagation (i.e., not re-entrant) in a detailed 3D model of a human left ventricular wall slab, which manifests as significant differences in the body surface potential activation maps (Figure 10C). The advantages of using detailed 3D models (in this case, where the geometry is obtained from diffusion tensor MRI) lie in their ability to reproduce the orthotropic conduction velocities resulting from complicated tissue architecture (i.e., fiber and sheet structure; Benson et al., 2007; Smaill et al., 2013) and the boundary and curvature effects that can modulate electrotonic coupling and propagation (Walton et al., 2013; Campos et al., 2015): these effects are crucial in understanding the complex and chaotic propagation patterns underlying cardiac arrhythmias. Nevertheless, the 1D models used in this study allow us to examine arrhythmia trigger-substrate interaction in a simple and methodological manner.

### Limitations

In this study, a general model of intracellular Ca^2+^ handling was integrated with a simplified formulation of a human ventricular AP model. Due to the general nature of the Ca^2+^ handling model, details of heterogeneity in Ca^2+^ handling were not included in order to avoid introducing artifacts. For this reason, and due to the multi-scale focus of the study, detailed investigation of the mechanisms of spontaneous release in single cell, and their regional dependencies, was not performed, and investigation was instead limited to the potential effect of MC-II-157c on reversing SR Ca^2+^ loading. There are many possible mechanisms of spontaneous release associated with diseases linked to prolonged QT interval (e.g., hyper phosphorylation of the RyRs; upregulation of SERCA; detubulation) which were not considered in the present study. However, the aim of this study was to investigate the multi-scale interaction between trigger and substrate, and the use of the simplified spontaneous release functions in single-cell and tissue simulations allowed this to be analyzed in a general manner and across a large range of conditions, independent of spontaneous release mechanism. In future, combining detailed single cell studies of the mechanisms of spontaneous release in disease conditions with tissue simulations of the same conditions would provide further mechanistic insight; a dynamic simplified spontaneous release model would furthermore allow the study of long-term interactions between trigger and substrate in tissue e.g., during re-entry.

The factors underlying the synchronization and propagation of ectopic activity are highly complex and it is therefore worth making explicit that our simplified approach (which does not consider, for example, heterogeneity in the distributions describing Ca^2+^ release) is primarily suitable for interpretation of general trends only, rather than as a quantitative analysis of the efficacy of MC-II-157c in modulating Ca^2+^ release dependent triggers; it is encouraging to note that our results are consistent with those of a previous study (Campos et al., 2015), with the emergence of premature excitation from independent stochastic events overcoming electrotonic load, and the steep, “all-or-nothing” relationship observed in tissue. This steep relationship also likely accounts for the lack of effect of MC-II-157c on PQTb (the condition exhibiting the highest vulnerability to ectopic activity), wherein the distributions selected were not close enough to the threshold region to reveal an effect.

The relative contribution of *I*_Kr_ to repolarisation is different in different models of the human ventricular action potential (Mirams et al., 2014): for example, *I*_Kr_ contributes more repolarising current in the ORd model (O'Hara et al., 2011) than in the ten Tusscher & Panfilov model (ten Tusscher and Panfilov, 2006), despite both models being validated against experimental data. It is therefore possible that our results overestimate the effects of the hERG activator MC-II-157c, although the identified pro- and anti-arrhythmic mechanisms will still be relevant.

The limitations of using 1D and 2D simplifications of 3D cardiac tissue have been discussed in detail previously (Clayton et al., 2011). Here we only note that the 1D strand model of the left ventricular wall allows us to examine mechanisms underlying how arrhythmia triggers and substrates interact to initiate unidirectional propagation, without the added complicating effects that geometrical (shape) and architectural (fiber, sheet etc.) considerations would bring. Nevertheless, it is anticipated that these geometrical and architectural effects will play a role not only in the transition from unidirectional propagation to re-entry, but on the initiation of the unidirectional propagation itself through, for example, electrotonic effects (Benson et al., 2007; Walton et al., 2013). Similarly, our 2D and 3D tissues were isotropic, i.e., no fiber or sheet structure, and so any conclusions drawn from these simulations should be interpreted with this in mind. Elucidating the roles that tissue geometry and architecture play in arrhythmogenesis (Smaill et al., 2013) is an important next step in fully understanding arrhythmia trigger and substrate interaction.

The spatial distributions of endocardial, midmyocardial, and epicardial cell types across the human ventricular wall has still not been confirmed: some studies suggest that midmyocardial cells are found predominantly in isolated regions of the subendocardium (Glukhov et al., 2010), while others suggest a continuous population of midmyocardial cells in the subepicardial region (Drouin et al., 1995); these distributions may be dependent on species, location in the ventricular wall, and disease state (Antzelevitch, 2010; Strom et al., 2010). Because of this uncertainty, we set the spatial distribution of the three cell types to be equal in the transmural direction, but the effects of the electrotonic interactions of different regions of cell types are likely to be qualitatively similar if these distributions are altered.

The simulated model of MC-II-157c reproduced qualitatively the key features of the effect of the compound on *I*_Kr_ (i.e., an increased activity during a depolarising pulse), but it is important to note that there were significant differences between the simulation and experimental data (Figure 5): firstly, the formulation of *I*_Kr_ implemented does not have a time-dependent inactivation, which is observed in the experimental trace; secondly, the simulated data exhibited a larger difference in the magnitude of the current during the depolarisation step between baseline and MC-II-157c than observed experimentally. A more detailed model of *I*_Kr_ in both basal and MC-II-157c conditions would be essential for future and more detailed analysis of the compound specifically.

Furthermore, recent work, (e.g., Li et al., 2017), has highlighted that simple modulation of Hodgkin-Huxley current formulations, as used to simulate *I*_Kr_ in the ORd model, may not sufficiently capture complex drug-channel interaction dynamics, and that more complex Markov model formulations may be necessary to simulate such dynamics. However, until a full experimental characterisation of the dynamics of MC-II-157c effects on *I*_Kr_ under a range of conditions is carried out, allowing a validated Markov model of drug-channel interactions to be developed, we make use of the available data (Guo et al., 2014) to modify the Hodgkin-Huxley formulation used in the ORd model. The effects of MC-II-157c on *I*_Na_ are not as well characterized as its effects on *I*_Kr_ (Guo et al., 2014). We simulated the drug's action on *I*_Na_ by a simple reduction in the maximal conductance of the current. It is possible, however, that MC-II-157c also modifies the current's kinetics (i.e., shifts to the current's activation and inactivation curves, and/or a change to the time constants associated with these processes) in a similar manner to the way in which the kinetics of *I*_Kr_ are modified. Thus, further experimental characterisation of the drug's effects on both *I*_Kr_ and *I*_Na_ are required.

## Conclusion

The relative balance of reduced trigger and increased substrate underlies a multi-dimensional role of MC-II-157c in modulation of arrhythmia vulnerability associated with prolonged QT interval. Our results highlight that studies examining the efficacy of putative anti-arrhythmic drugs need to assess the effects of the drug on both the triggers and the substrates involved in arrhythmogenesis, i.e., such studies should adopt a multiscale approach to examine both cell- and tissue-level effects.

## Author contributions

All authors conceived and designed the study; MC, EP, and AB carried out simulations, and acquired and analyzed data; all authors interpreted the data; MC and AB prepared the first draft of the manuscript text; MC and EP prepared the figures; all authors edited the manuscript; all authors approved the final version of the manuscript; all authors agree to be accountable for all aspects of the work in ensuring that questions related to the accuracy or integrity of any part of the work are appropriately investigated and resolved.

### Conflict of interest statement

The authors declare that the research was conducted in the absence of any commercial or financial relationships that could be construed as a potential conflict of interest. The reviewer KF and handling Editor declared their shared affiliation.
