# Supplementary material for: Trigger vs. Substrate: Multi-Dimensional Modulation of QT-Prolongation Associated Arrhythmic Dynamics by a hERG Channel Activator
Source: Front Physiol. 2017 Oct 4;8:757. doi: 10.3389/fphys.2017.00757 (PMC5632683; doi:10.3389/fphys.2017.00757)
Supplement: Supplementary file 1 [file DataSheet1.pdf]

ONLINE SUPPLEMENT

Trigger versus substrate: multi-dimensional modulation of QT-prolongation associated arrhythmic dynamics by a hERG channel activator

Michael A. Colman[1]*, Erick A. Perez Alday[2], Arun V. Holden[1], Alan P. Benson[1]

[1]School of Biomedical Sciences & Multidisciplinary Cardiovascular Research Centre, University of Leeds, Leeds, UK

[2]Division of Cardiovascular Medicine, Oregon Health and Science University, Portland, Oregon, USA

Correspondence:
Michael A. Colman
m.a.colman@leeds.ac.uk

Contents

Spatial Ca^{2+} cycling model 3
Variables 3
Fundamental equations for Ca^{2+} concentration 4
Reaction terms 5
Intracellular Ca^{2+} release, J_{rel} 5
L-type Ca^{2+} channel flux, J_{CaL} 6
Intracellular uptake and leak, J_{up} and J_{leak} 9
Membrane fluxes, J_{NaCa}, J_{pCa} and J_{CaB} 9
Ca^{2+} Buffering 10
Instantaneous buffering in the cytoplasm 10
Instantaneous buffering in the jSR 10
References 11

Spatial Ca^{2+} cycling model

This section describes the spatial Ca^{2+} cycling model which has been integrated with the ventricular AP model. This model is a simplified and efficient version of a previously developed model [1].

Variables

Table S1: Ca^{2+} handling model variables

Parameter	Description	Unit
$[Ca^{2+}]_{ds}$	Average Ca^{2+} concentration in the dyadic cleft	µM
$^m[Ca^{2+}]_{ds}$	Ca^{2+} concentration in dyad in CRU *m*	µM
$[Ca^{2+}]_{rbSS}$	Average Ca^{2+} concentration in the subspace	µM
$^m[Ca^{2+}]_{rbSS}$	Ca^{2+} concentration in subspace in CRU *m*	µM
$[Ca^{2+}]_{cyto}$	Average Ca^{2+} concentration in the bulk cytoplasm	µM
$^m[Ca^{2+}]_{cyto}$	Ca^{2+} concentration in cytoplasm in CRU *m*	µM
$[Ca^{2+}]_{nSR}$	Average Ca^{2+} concentration in the network SR	µM
$^m[Ca^{2+}]_{nSR}$	Ca^{2+} concentration in network SR in CRU *m*	µM
$[Ca^{2+}]_{jSR}$	Average Ca^{2+} concentration in the junctional SR	µM
$^m[Ca^{2+}]_{jSR}$	Ca^{2+} concentration in junctional SR in CRU *m*	µM
$^mJ_{rel}$ | J_{rel}	Release flux in dyad *m* | Whole cell average release flux	$\mu M.ms^{-1}$
$^mJ_{CaL}$ | I_{CaL}	LTCC flux in dyad *m* | Whole cell current	$\mu M.ms^{-1}$ | pA/pF
$^mJ_{up}$ | J_{up}	SR Uptake flux in CRU *m* | Whole cell SR update flux	$\mu M.ms^{-1}$
$^mJ_{leak}$ | J_{leak}	SR leak flux in CRU *m* | Whole cell SR leak flux	$\mu M.ms^{-1}$
$^mJ_{ds}$	Flux from dyad *m* to rbSS voxel at *m*	$\mu M.ms^{-1}$
$^mJ_{ss}$	Flux from rbSS to cytoplasm voxel *m*	$\mu M.ms^{-1}$
$^mJ_{jsr}$	Flux from network to junctional SR at dyad *m*	$\mu M.ms^{-1}$
$^mJ_{trpn}$	Trpn buffering flux at CRU *m*	$\mu M.ms^{-1}$
$^mJ_{NaCa}$ | I_{NaCa}	Sodium-Ca^{2+} exchanger flux in CRU *m* | Whole cell current	$\mu M.ms^{-1}$ | pA/pF
$^mJ_{pCa}$ | I_{pCa}	PMCA Ca^{2+} pump flux in in CRU *m* | Whole cell current	$\mu M.ms^{-1}$ | pA/pF
J_{Cab} | I_{Cab}	Background Ca^{2+} current flux in voxel *p* | Whole cell current	$\mu M.ms^{-1}$ | pA/pF
$^m\beta_{cyto}$	Instantaneous buffering in the cytoplasm in CRU *m*	-
$^m\beta_{jSR}$	Instantaneous buffering in the junctional SR in CRU *m*	-
$^mn_{o\_ryr}$	Number of open RyRs in dyad *m*	-
^mCA	Number of RyRs in the Closed Activated state, dyad *m*	-
^mOA	Number of RyRs in the Open Activated state, dyad *m*	-
^mCI	Number of RyRs in the Closed Inactivated state, dyad *m*	-
^mOI	Number of RyRs in the Open Inactivated state, dyad *m*	-
csqn	Free calsequestrin concentration	mM
^mM	Proportion of csqn in monomer state, dyad *m*	-
$^md_1$	LTCC activation gate state 1, dyad *m*	-
$^md_2$	LTCC activation gate state 2, dyad *m*	-
$^md_3$	LTCC activation gate state 3, dyad *m*	-
$^mf_1$	LTCC voltage-dependent inactivation state 1, dyad *m*	-
$^mf_2$	LTCC voltage-dependent inactivation state 2, dyad *m*	-
$^mfca_1$	LTCC Ca^{2+}-dependent inactivation state 1, dyad *m*	-
$^mfca_2$	LTCC Ca^{2+}-dependent inactivation state 2, dyad *m*	-
V_m	Membrane potential	mV

Fundamental equations for Ca^{2+} concentration

$$\frac{d[Ca^{2+}]_i}{dt} = \beta_i \left(\mathbf{D}\nabla^2[Ca^{2+}]_i + \phi_i + \left(v_{ss}/v_i\right) J_{ss}\right) \tag{1}$$

$$\frac{d[Ca^{2+}]_{SS}}{dt} = \mathbf{D}\nabla^2[Ca^{2+}]_{SS} + \phi_{SS} - J_{ss} + \left(v_{ds}/v_{ss}\right) J_{ds} \tag{2}$$

$$\frac{d[Ca^{2+}]_{nSR}}{dt} = \beta_{nSR} \left(\mathbf{D}\nabla^2[Ca^{2+}]_{nSR} + \phi_{nSR} - \left(v_{jsr}/v_{nsr}\right) J_{jSR}\right) \tag{3}$$

$$\frac{d\left[Ca^{2+}\right]_{ds}}{dt} = \phi_{ds} - J_{ds} \tag{4}$$

$$\frac{d\left[Ca^{2+}\right]_{JSR}}{dt} = \phi_{JSR} + J_{jSR} \tag{5}$$

Where transfer between compartments is given by:

$$J_{ss} = \left(\left[Ca^{2+}\right]_{SS} - \left[Ca^{2+}\right]_i\right)\tau_{ss}^{-1} \tag{6}$$

$$J_{ds} = \left(\left[Ca^{2+}\right]_{ds} - \left[Ca^{2+}\right]_{SS}\right)\tau_{ds}^{-1} \tag{7}$$

$$J_{jSR} = \left(\left[Ca^{2+}\right]_{JSR} - \left[Ca^{2+}\right]_{nSR}\right)\tau_{jSR}^{-1} \tag{8}$$

And the reaction terms are:

$$\phi_i = J_{NaCa} + J_{pCa} + J_{Cab} - \left(J_{up} - J_{leak}\right) - J_{trpn} \tag{9}$$

$$\phi_{nSR} = \left(J_{up} - J_{leak}\right)\left(v_i/v_{nsr}\right) \tag{10}$$

$$\phi_{ss} = J_{NaCa\_SS} \tag{11}$$

$$\phi_{ds} = J_{rel} + J_{CaL} \tag{12}$$

$$\phi_{JSR} = -J_{rel}\left(v_{ds}/v_{jSR}\right) \tag{13}$$

Spatial diffusion is described by a 6-node nearest neighbours finite difference approximation:

$$\mathbf{D}\nabla^2[Ca^{2+}]_x \approx J_{Ca\_diff\_x} = \sum_{i=1}^{i=3}\left(\frac{{}^{e_i+1}[Ca^{2+}]_x + {}^{e_i-1}[Ca^{2+}]_x - {}^{e_i}[Ca^{2+}]_x}{\tau_{x,e_i}}\right) \tag{14}$$

Where e_i refers to the three dimensions (x,y,z)

Table S2: Cell structure and diffusion parameters

Parameter	Description	Value
v_i	Cytoplasm volume per CRU	1.0 μm^3
v_{nsr}	Network SR volume per CRU	0.05 μm^3
v_{SS}	Sub-space volume per CRU	0.0175 μm^3
$<v_{ds}>$	Average volume of individual dyad	1.712 x10^{-3} μm^3
v_{jSR}	Volume individual jSR	0.015 μm^3
τ_{ds}	Time constant diffusion from dyad to SS	0.022 ms
τ_{ss}	Time constant diffusion from SS to cytoplasm	0.1 ms
τ_{jSR}	Time constant diffusion from nSR to jSR	5 ms
$\tau_{i,transverse}$	Time constant of transverse cytoplasm diffusion	2.3 ms
$\tau_{i,longitudinal}$	Time constant of longitudinal cytoplasm diffusion	2.9 ms
$\tau_{ss,transverse}$	Time constant of transverse subspace diffusion	1.25 ms
$\tau_{ss,longitudinal}$	Time constant of longitudinal subspace diffusion	1.95 ms
$\tau_{nSR,transverse}$	Time constant of transverse nSR diffusion	7 ms
$\tau_{nSR,longitudinal}$	Time constant of longitudinal nSR diffusion	14 ms

Reaction terms

The formulations for the reaction terms (Equations (9-13)) are described below:

Intracellular Ca^{2+} release, J_{rel}

$$^{m}J_{rel} = {}^{m}k_{rel}\left({}^{m}\left[Ca^{2+}\right]_{jSR} - {}^{m}\left[Ca^{2+}\right]_{ds}\right) \tag{15}$$

$$^{m}k_{rel} = {}^{m}n_{o\_RyR} . g_{RyR} . {}^{m}v_{ds}^{-1} \tag{16}$$

$^{m}n_{o\_RyR}$ is the number of open RyR channels in dyad *m*. RyR dynamics is described by a 4-state Markov Chain model. The model is similar to Stern et al [2] and Restrepo et al [3], with a functional monomer induced inactivation based on csqn dynamics described by Gaur-Rudy [4].

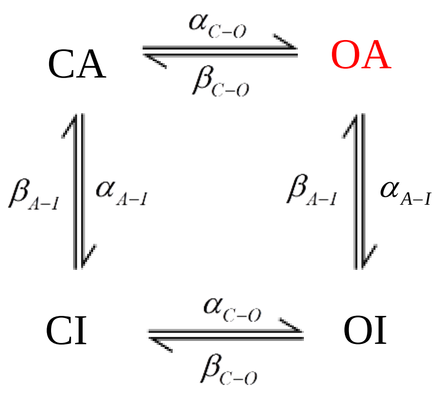

$$\frac{d\,{}^{m}CA}{dt} = {}^{m}OA.\beta_{C-O} + {}^{m}CI.\beta_{A-I} - {}^{m}CA.(\alpha_{C-O} + \alpha_{A-I}) \tag{17}$$

$$\frac{d\,{}^{m}OA}{dt} = {}^{m}CA.\alpha_{C-O} + {}^{m}OI.\beta_{A-I} - {}^{m}OA.(\beta_{C-O} + \alpha_{A-I}) \tag{18}$$

$$\frac{d\,{}^{m}CI}{dt} = {}^{m}OI.\beta_{C-O} + {}^{m}C.\alpha_{A-I} - {}^{m}CI.(\alpha_{C-O} + \beta_{A-I}) \tag{19}$$

$$\frac{d\,{}^{m}OI}{dt} = {}^{m}CI.\alpha_{C-O} + {}^{m}OA.\alpha_{A-I} - {}^{m}OI.(\beta_{C-O} + \beta_{A-I}) \tag{20}$$

Where:

$$\alpha_{C-O} = k_a \left({}^{m}\left[Ca^{2+}\right]_{ds} \right)^{H} \tag{21}$$

$$\beta_{C-O} = k_b \tag{22}$$

$$\alpha_{A-I} = \left(1 - {}^{m}Mi_{ss}\right) / \tau_{Mi,1} \tag{23}$$

$$\beta_{A-I} = {}^{m}Mi_{ss} / \tau_{Mi,2} \tag{24}$$

$${}^{m}Mi_{ss} = 1 \Big/ \left(1 + e^{({}^{m}M - 0.5)/0.04167}\right) \tag{25}$$

$$\frac{dM}{dt} = \alpha_M (1 - M) + \beta_M M \tag{26}$$

$$\alpha_M = M_{ss} / \tau_{M,1} \tag{27}$$

$$\beta_M = (1 - M_{ss}) / \tau_{M,2} \tag{28}$$

$$M_{ss} = 1 / (1 + e^{(-6.5.(csqn - 6.37))}) \tag{29}$$

$$csqn = B_{csqn}.K_{mcsqn} \Big/ \left({}^{m}\left[Ca^{2+}\right]_{jSR} + K_{mcsqn} \right) \tag{30}$$

In this model, *OA* is the only state in which a flux occurs. Thus, ${}^{m}n_{o\_RyR}$ is equal to the number of channels in dyad *m* which are in state *OA* (red text in schematic).

<u>L-type Ca^{2+} channel flux,</u> J_{CaL}

The flux through the L-type Calcium Current is defined as:

$${}^{m}J_{CaL} = -{}^{m}n_{o\_LTCC}\,{}^{m}\bar{J}_{CaL} \tag{31}$$

Where ${}^{m}n_{o\_LTCC}$ is the number of open LTCC channels in dyad *m* (defined below) and $\bar{J}_{CaL}$ is the maximal flux rate per channel [5]:

$${}^{m}\bar{J}_{CaL} = 4P_{Ca}zF\,\frac{\frac{1}{2}\gamma_{Ca}\,{}^{m}\left[Ca^{2+}\right]_{ds} e^{2z} - \gamma_{Ca}\left[Ca^{2+}\right]_{o}}{e^{2z} - 1} \tag{32}$$

$$z = \frac{V_m F}{RT} \tag{33}$$

Where $[Ca^{2+}]_o$ is the extracellular Ca^{2+} concentration, P_{Ca} is the maximum permeability of an individual LTCC and *F* is the Faraday constant.

The LTCCs are described by a Markov Chain construction of a Hodkin-Huxley model [5,6]

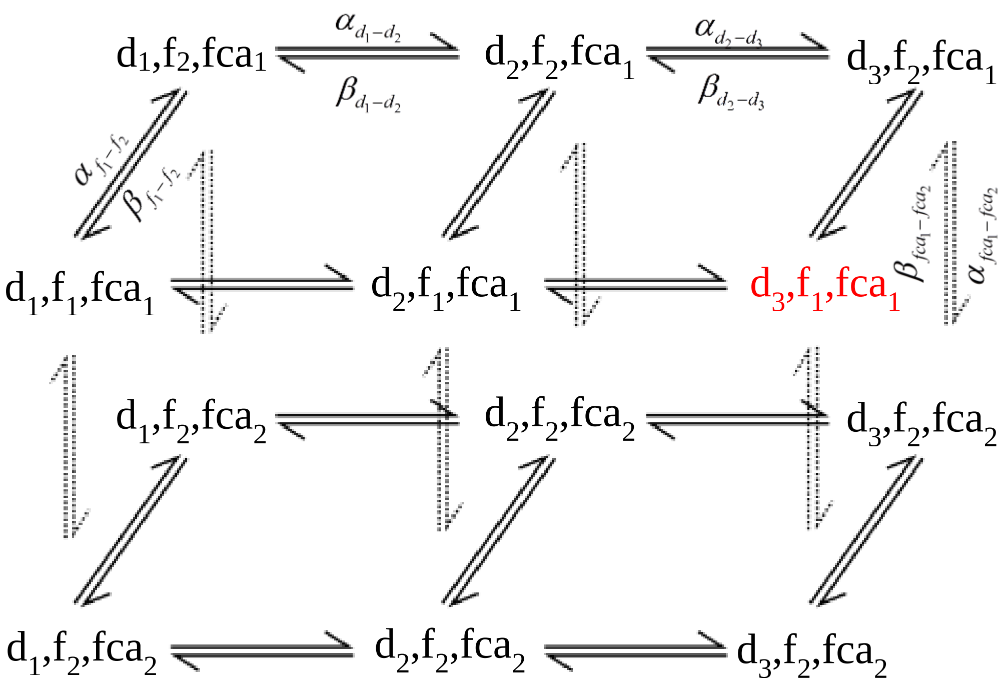

Which is equivalent to the three gate Hodgkin-Huxley model:

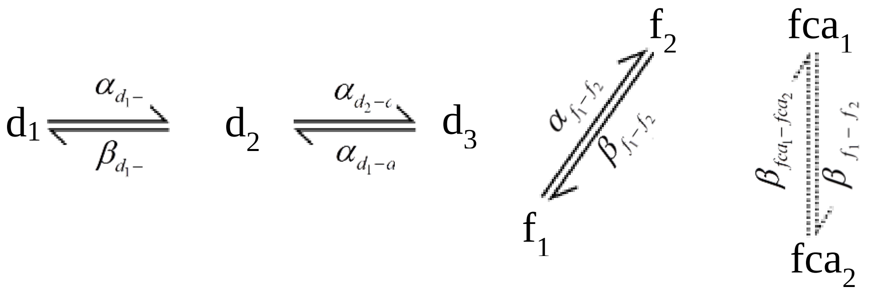

And thus described by:

$$\frac{d(d_1)}{dt} = d_2\beta_{d_1-d_2} - d_1\alpha_{d_1-d_2} \tag{34}$$

$$\frac{d(d_2)}{dt} = d_1\alpha_{d_1-d_2} + d_3\beta_{d_2-d_3} - d_2(\beta_{d_1-d_2} + \alpha_{d_2-d_3}) \tag{35}$$

$$\frac{d(d_3)}{dt} = d_2\alpha_{d_2-d_3} - d_3\beta_{d_2-d_3} \tag{36}$$

$$\frac{d(f_1)}{dt} = f_2\beta_{f_1-f_2} - f_1\alpha_{f_1-f_2} \tag{37}$$

$$\frac{d(fca_1)}{dt} = fca_2\beta_{fca_1-fca_2} - fca_1\alpha_{fca_1-fca_2} \tag{38}$$

Where the transition rates for each variable couplet, (x = d_1-d_2, f_1-f_2, fca_1-fca_2) are defined from the steady-state and time constant in the standard way:

$$\alpha_x = x_{ss} / \tau_x \tag{39}$$

$$\beta_x = (1 - x_{ss}) / \tau_x \tag{40}$$

And:

$$\alpha_{d_2-d_3} = k_{d2d3} \tag{41}$$

$$\beta_{d_2-d_3} = k_{d3d2} \tag{42}$$

$$d_{ss} = 1 \Big/ \left(1 + e^{(-(V_m - 5)/6.24)}\right) \tag{43}$$

$$f_{ss} = 1 - 1 \Big/ \left(1 + e^{((V_m + 32.06)/8.6)}\right) \tag{44}$$

$$\tau_d = d_{ss} . \left(1 - e^{(-(V_m - 5)/6.24)}\right) \Big/ \left(0.035(V_m - 5)\right) \tag{45}$$

$$\tau_f = 2 \Bigg/ \left(0.0197 e^{-\left(\left[0.0337(V_m + 7)\right]^2 + 0.02\right)}\right) \tag{46}$$

$$fca_{ss} = 1 - 1 \Bigg/ \left(1 + \left({}^{m}\left[Ca^{2+}\right]_{ds} \Big/ \bar{C}a\right)^2\right) \tag{47}$$

Note that the steady states of the inactivation gates (*f, fca*) are inverse to those in the standard Hodgkin-Huxley model because in this Markov description f_2 is the inactivated state, equivalent to (*1-f)* in the standard description (and f_1 is equivalent to *f*).

Table S3: RyR and LTCC flux parameters

Parameter	Description	Value
g_{ryr}	Maximal flux rate through the RyRs	2.05 x 10^{-4} $\mu m^3\ ms^{-1}$
P_{Ca}	Maximum permeability of LTCC	11.9 $\mu mol\ C^{-1}\ ms^{-1}$
γ_{Ca}	Activity Coefficient LTCC	0.341
N_{RyR}	Number of RyRs per dyad*	100
H	RyR Open rate Ca^{2+} power	2.5
k_a	RyR activation rate constant	1.58 x 10^{-4} $\mu M^{-2.5}\ ms^{-1}$
k_b	RyR deactivation rate constant	1.0 ms^{-1}
$\tau_{M,1}$	Time constant of monomer binding	25 ms
$\tau_{Mi,1}$	Time constant of monomer inactivation	30 ms
$\tau_{M,2}$	Time constant monomer unbinding	156 ms
$\tau_{Mi,2}$	Time constant of de-inactivation	75 ms
N_{LTCC}	Number of L-type Ca^{2+} channels per dyad*	15
k_{d2d3}	Rate constant for transition d_2-d_3	0.3 ms^{-1}
k_{d3d2}	Rate constant for transition d_3-d_2	6.0 ms^{-1}
τ_{fca}	Time constant for Ca^{2+} induced inactivation	15 ms
$\bar{C}a$	Ca^{2+} constant for Ca^{2+} induced inactivation	6.0 μM
$[Ca^{2+}]_o$	Extracellular Ca^{2+} concentration	1.8 mM

Intracellular uptake and leak, J_{up} and J_{leak}

These equations are based on Restrepo et al [3] and preceding studies [7,8].

$$ {}^{m}J_{up} = g_{up}\frac{\left({}^{m}\left[Ca^{2+}\right]_i \big/ K_{cyto}\right)^2 - \left({}^{m}\left[Ca^{2+}\right]_{nSR} \big/ K_{nSR}\right)^2}{1+\left({}^{m}\left[Ca^{2+}\right]_i \big/ K_{cyto}\right)^2 + \left({}^{m}\left[Ca^{2+}\right]_{nSR} \big/ K_{nSR}\right)^2} \tag{48} $$

$$ {}^{m}J_{leak} = g_{leak}\frac{{}^{m}\left[Ca^{2+}\right]_{nSR}^2}{{}^{m}\left[Ca^{2+}\right]_{nSR}^2 + K_{leak}^2}\left({}^{m}\left[Ca^{2+}\right]_{nSR} - {}^{m}\left[Ca^{2+}\right]_i\right) \tag{49} $$

Table S4: Ca^{2+} uptake and leak parameters

Parameter	Description	Value
g_{up}	Maximal flux rate of J_{up}	0.339 µM.ms^{-1}
K_{cyto}	Cytoplasm constant for J_{up}	0.15 µM
K_{nSR}	Network SR constant for J_{up}	1700 µM
g_{leak}	Maximal flux rate of J_{leak}	1.412 x10^{-5} ms^{-1}
K_{leak}	J_{leak} constant	450 µM

Membrane fluxes, J_{NaCa}, J_{pCa} and J_{CaB}

$$ {}^{m}J_{NaCa} = \frac{K_a g_{NaCa} v_{vox}^{-1}\left(e^{\eta z}\left[Na^+\right]_i\left[Ca^{2+}\right]_o - e^{(\eta-1)z}\left[Na^+\right]_o {}^{m}\left[Ca^{2+}\right]_{cyto}\right)}{(t_1+t_2+t_3)\left(1+K_{sat}e^{(\eta-1)z}\right)} \tag{50} $$

$$ {}^{m}J_{pCa} = \left(v_i^{-1} g_{pCa}\, {}^{m}\left[Ca^{2+}\right]_i\right) \Big/ \left(K_{mpCa} + {}^{m}\left[Ca^{2+}\right]_i\right) \tag{51} $$

$$ {}^{m}J_{Cab} = v_{vox}^{-1} g_{Cab}\left(V_m - E_{r,Ca}\right) \tag{52} $$

Where

$$ t_1 = K_{mCai}\left[Na^+\right]_o^3\left(1+\left(\left[Na^+\right]_i \big/ K_{mNai}\right)^3\right) \tag{53} $$

$$ t_2 = K_{mNao}^3\, {}^{m}\left[Ca^{2+}\right]_i\left(1+\left({}^{m}\left[Ca^{2+}\right]_i \big/ K_{mCai}\right)\right) \tag{54} $$

$$ t_3 = K_{mCao}\left[Na^+\right]_i^3 + \left[Na^+\right]_i^3\left[Ca^{2+}\right]_o + \left[Na^+\right]_o^3\, {}^{m}\left[Ca^{2+}\right]_i \tag{55} $$

$$ K_a = \left[1+\left(K_{da} \big/ {}^{m}\left[Ca^{2+}\right]_i\right)\right] \tag{56} $$

$$ z = \frac{V_m F}{RT} \tag{57} $$

Table S5: Membrane flux parameters

Parameter	Description	Value
g_{NaCa}	Maximal flux rate of Sodium-Ca^{2+} exchanger	0.3726 $\mu m^3.\mu M.ms^{-1}$
K_{da}	Ca^{2+} scaling constant	0.11 µM
η	Voltage sensitivity coefficient	0.35
K_{sat}	Saturation constant	0.27
K_{mcai}	Intracellular Ca^{2+} constant	3.59 µM
K_{mcao}	Extracellular Ca^{2+} constant	1.3 mM
K_{mnai}	Intracellular Na^+ constant	12.3 mM
K_{mnao}	Extracellular Na^+ constant	87.5 mM
g_{pca}	Maximal flux rate of PMCA Ca^{2+} pump	1.37 $x10^{-3}$ $\mu m^{3}\cdot\mu M.ms^{-1}$
g_{cab}	Maximal flux rate of background Ca^{2+} current	1.82 $x10^{-5}$ $\mu m^3.\mu M.ms^{-1}.mV^{-1}$
$[Na^+]_i$	Intracellular Na^+ concentration	7.95 mM
$[Na^+]_o$	Extracellular Na^+ concentration	136 mM

Ca^{2+} Buffering

Instantaneous buffering in the cytoplasm

Instantaneous buffering in the cytoplasm follows that of previous models, e.g., Restrepo et al [3] and Nivala et al. [9], based on [10]. At each voxel, *n*, the buffering term is given by:

$$ {}^{m}\beta_{cyto} = \left[1 + \sum_{x} \frac{B_x K_x}{\left({}^{m}[Ca^{2+}]_{cyto} + K_x \right)^2} \right]^{-1} \tag{58} $$

Where *x* refers to four buffering processes: Calmodulin, SR sites, Myosin (Ca) and Myosin (Mg).

Instantaneous buffering in the jSR

Buffering in the jSR follows that of the previous study Gaur-Rudy [4]:

$$ {}^{m}\beta_{jSR} = \left[1 + \frac{B_{csqn} K_{mcsqn}}{\left({}^{m}[Ca^{2+}]_{jSR} + K_{mcsqn} \right)^2} \right]^{-1} \tag{59} $$

Table S4: Ca^{2+} buffering parameters

Parameter	Description	Value
K_{CAM}	Dissociation constant for calmodulin	7.0 µM
B_{CAM}	Total concentration buffering sites	24.0 µM
K_{SR}	Dissociation constant for SR sites	0.6 µM
B_{SR}	Total concentration buffering sites	47.0 µM
$K_{M,Ca}$	Dissociation constant for Myosin (Ca)	0.033 µM
$B_{M,Ca}$	Total concentration buffering sites	140.0 µM
$K_{M,Mg}$	Dissociation constant for Myosin (Mg)	3.64 µM
$B_{M,Mg}$	Total concentration buffering sites	140 µM
K_{mcsqn}	Dissociation constant for csqn	0.8 mM
B_{csqn}	Total concentration buffering sites	10 mM

References

1. Colman MA, Pinali C, Trafford AW, Zhang H, Kitmitto A. A computational model of spatio-temporal cardiac intracellular calcium handling with realistic structure and spatial flux distribution from sarcoplasmic reticulum and t-tubule reconstructions. PLOS Comput Biol. 2017 Aug 31;13(8):e1005714.

2. Stern MD, Song LS, Cheng H, Sham JS, Yang HT, Boheler KR, et al. Local control models of cardiac excitation-contraction coupling. A possible role for allosteric interactions between ryanodine receptors. J Gen Physiol. 1999 Mar;113(3):469–89.

3. Restrepo JG, Weiss JN, Karma A. Calsequestrin-mediated mechanism for cellular calcium transient alternans. Biophys J. 2008 Oct;95(8):3767–89.

4. Gaur N, Rudy Y. Multiscale modeling of calcium cycling in cardiac ventricular myocyte: macroscopic consequences of microscopic dyadic function. Biophys J. 2011 Jun 22;100(12):2904–12.

5. Luo CH, Rudy Y. A dynamic model of the cardiac ventricular action potential. I. Simulations of ionic currents and concentration changes. Circ Res. 1994 Jun;74(6):1071–96.

6. Song Z, Ko CY, Nivala M, Weiss JN, Qu Z. Calcium-voltage coupling in the genesis of early and delayed afterdepolarizations in cardiac myocytes. Biophys J. 2015 Apr 21;108(8):1908–21.

7. Shiferaw Y, Watanabe MA, Garfinkel A, Weiss JN, Karma A. Model of intracellular calcium cycling in ventricular myocytes. Biophys J. 2003 Dec;85(6):3666–86.

8. Shannon TR, Wang F, Puglisi J, Weber C, Bers DM. A mathematical treatment of integrated Ca dynamics within the ventricular myocyte. Biophys J. 2004 Nov;87(5):3351–71.

9. Nivala M, de Lange E, Rovetti R, Qu Z. Computational modeling and numerical methods for spatiotemporal calcium cycling in ventricular myocytes. Front Physiol. 2012;3:114.

10. Wagner J, Keizer J. Effects of rapid buffers on Ca2+ diffusion and Ca2+ oscillations. Biophys J. 1994 Jul;67(1):447–56.
